# Mapping and characterization of G-quadruplexes in the genome of the social amoeba *Dictyostelium discoideum*

**DOI:** 10.1093/nar/gkz196

**Published:** 2019-03-29

**Authors:** Mona Saad, Aurore Guédin, Souheila Amor, Amina Bedrat, Nicolas J Tourasse, Hussein Fayyad-Kazan, Geneviève Pratviel, Laurent Lacroix, Jean-Louis Mergny

**Affiliations:** 1 ARNA Laboratory, IECB, Inserm U1212, CNRS UMR 5320, Université de Bordeaux, Bordeaux, France; 2 Laboratory of Cancer Biology and Molecular Immunology, Faculty of Sciences I, Lebanese University, Beirut, Al-Hadath, Lebanon, Lebanon; 3 Laboratoire de Chimie de Coordination, CNRS UPR 8241, Toulouse, France; 4 Inserm U1024, CNRS UMR 8197, IBENS, Paris, France; 5 Institute of Biophysics of the Czech Academy of Sciences, v.v.i., Královopolská 135, 612 65 Brno, Czech Republic

## Abstract

G-quadruplexes (G4) are non-canonical DNA and/or RNA secondary structures formed in guanine-rich regions. Given their over-representation in specific regions in the genome such as promoters and telomeres, they are likely to play important roles in key processes such as transcription, replication or RNA maturation. Putative G4-forming sequences (G4FS) have been reported in humans, yeast, bacteria, viruses and many organisms. Here we present the first mapping of G-quadruplex sequences in *Dictyostelium discoideum*, the social amoeba. ‘Dicty’ is an ameboid protozoan with a small (34 Mb) and extremely AT rich genome (78%). As a consequence, very few G4-prone motifs are expected. An *in silico* analysis of the *Dictyostelium* genome with the G4Hunter software detected 249–1055 G4-prone motifs, depending on G4Hunter chosen threshold. Interestingly, despite an even lower GC content (as compared to the whole Dicty genome), the density of G4 motifs in *Dictyostelium* promoters and introns is significantly higher than in the rest of the genome. Fourteen selected sequences located in important genes were characterized by a combination of biophysical and biochemical techniques. Our data show that these sequences form highly stable G4 structures under physiological conditions. Five *Dictyostelium* genes containing G4-prone motifs in their promoters were studied for the effect of a new G4-binding porphyrin derivative on their expression. Our results demonstrated that the new ligand significantly decreased their expression. Overall, our results constitute the first step to adopt *Dictyostelium discoideum* as a ‘G4-poor’ model for studies on G-quadruplexes.

## INTRODUCTION

G-quadruplex (G4) structures are non-canonical nucleic acids secondary structures that may form within guanine-rich regions. This family of DNA and/or RNA arrangements is characterized by the stacking of multiple G-quartets, where every G-quartet is a tetrad formed by the association of four guanine residues linked through Hoogsteen Hydrogen bonds and stabilized by monovalent cations, predominantly K^+^ (1). Under physiological conditions, G4 structures can fold into different topologies and can be formed by one, two, three or four strands. In addition to differences in molecularity, structural diversity depends on various parameters such as oligonucleotide sequence, strands orientation (parallel, antiparallel, hybrid), size and type of the loops, presence of bulges, non G-quartets, base pairs or triads, or the presence of specific ligands (2). The first report of a G-quartet motif dates back 56 years ago when a concentrated solution of guanylic acid GMP aggregated. After that, the research area about these structures expanded to cover different domains ranging from structural biology to nanotechnology (3).

The biological relevance of G4 structures has now been recognized thanks to a number of recent studies. According to *in silico* analyses of different genomes as well as *in vitro* assays, it is now well known that these structures are not distributed randomly in genomes but are enriched in key regions such as telomeres (5), oncogene promoters such as *c-myc* (6) or *KRAS* (7), immunoglobulin switch regions (8), and ribosomal DNA (9,10). Stable RNA G-quadruplexes were also demonstrated to be formed in 5′ UTRs (11), some introns (12) and in non-coding RNAs such as TERRA (13) and hTR (14,15). These findings suggest that G4 structures play important roles in a number of cellular processes. Since they are stable under physiological conditions, their presence may create a knot in the genome affecting replication, transcription and translation, and helicases may have a role in unwinding these structures to maintain the genetic stability during replication like the role of DOG1 (a FANCJ ortholog) in *Caenorhabditis elegans* (16) and Pif1 in yeast (17,18). Although the existence of G4 structures *in vivo* has been considered a state of debate, specific antibodies and *in vivo* Nuclear magnetic resonance (NMR) allowed the quantitative visualization of these structures in human cells (19–21).

G-quadruplexes represent attractive targets for anticancer drug design, as a number of oncogenes contain G4-prone motifs in their promoters and G4 ligands have antiproliferative effects on cancer cell lines and *in vivo* xenograft models. The development of G4-specific ligands as well as fluorescent probes to track these structures is a promising field in clinical diagnosis and fundamental research. To achieve this goal, accurate G4 prediction and better understanding of G4 topology *in vitro* are needed. Several algorithms were developed to predict putative G4-forming sequences (G4FS) including the commonly used Quadparser (22,23) and QGRS Mapper (24) tools and others. However, many biophysical studies revealed some shortcomings since a number of false negative and false positive sequences were found.

To that aim, we developed a new algorithm called G4Hunter that partially overcomes these weaknesses by taking into account G-richness and G-skewness and provides a propensity score for every tested sequence (25). G4Hunter accuracy was validated on over 500 sequences *in vitro*, including 200 candidates chosen from the human mitochondrial genome (16.6 kb) (25). It was then applied to genomes of a number of prokaryotes and eukaryotes including human. Our data allowed us to conclude that the number of putative G4FS in the human genome is significantly higher by a factor of 2–10 than previous estimates. One of the problems in studying G4 structures in the human genome is indeed the high number of putative G4FS (∼370 000 according to Quadparser; 500 000–700 000 according to G4-seq (26) and over 1 million when using a threshold of 1.5 with G4Hunter) (25). It is therefore difficult to deconvolute G4-related biological effects in human cells as many genes (50% or more) contain a G4-prone motif in their promoters and are therefore potential responders to G4 ligands.

A simpler G4-poor eukaryotic model would be helpful to complement human studies, in order to decrease the number of potential targets and facilitate deconvolution of biological effects at the transcriptome level. To that aim, we chose *Dictyostelium discoideum* as a simpler eukaryotic model. Putative G-quadruplex sequences (G4FS) have been found in organisms other than humans, such as yeast (27,28), bacteria (29), viruses (30) and parasites (31). However, to our knowledge, no systematic study of G4 structures in *Dictyostelium discoideum* has been reported.

*Dictyostelium discoideum* is a lower eukaryote, a slime mold that lives in the soil at moderate temperatures and feeds on yeast and bacteria. *D. discoideum* was approved by the National Institutes of Health as an important model organism (32). Because of its unique lifestyle characterized by the oscillation between unicellular and multicellular forms upon starvation, the social amoeba is an excellent organism used for studying cell motility, chemotaxis and signal transduction (33). In rich environment, *D. discoideum* cells grow as individual amoeba. However, upon starvation, a group of 10^5^ cells aggregate and enter a multicellular developmental process ending by the formation of fruiting bodies (34). *D. discoideum* genome is small with a length of 34 Mb, *i.e*., ≈100 times smaller than its human counterpart. It is therefore compact with few and short introns, as well as short non-coding intergenic sequences and is fully sequenced (35) and available in dictyBase (http://dictybase.org). Because of its high AT content (78%), *D. discoideum* represents an attractive model for G4 studies as far less G4 candidate sequences are expected.

In this work, G4 propensity was analyzed by using our algorithm G4Hunter, with an emphasis on *Dictyostelium* promoter sequences, as well as exons and introns. Due to their location in genes of key functions in *D. discoideum*, 14 sequences likely to form G4 structure were selected and analyzed by different biophysical methods. Our data demonstrated that all selected sequences form highly stable G4 structures under physiological conditions. In addition, a new porphyrin derivative affected the expression of five *Dictyostelium* genes with G4 sequences in their promoters. Our results suggest possible biological roles of quadruplexes in *D. discoideum* genome.

## MATERIALS AND METHODS

### Bioinformatics

Genome sequence (dicty_chromosomal.gz, created 13 May 2009 (35)) and annotation file (dicty_gff3.zip, generated 30 November 2016) for the *D. discoideum* strain AX4 were downloaded from dictyBase (dictybase.org). Only the six *Dictyostelium* chromosomes were considered for this study (extrachromosomal material was excluded). All bioinformatics procedures were conducted with R (v3.5.1) using the following packages: GenomicFeatures (v1.32.2), Biostrings (v2.48.0) and rtracklayer (v1.40.4) (36). G4 search was done by conserving the parameters used in our original G4Hunter publication (25): the difference here is that we restricted our search on a short and a large region relative to the annotated transcription start site (TSS) position, the first screening was done in the regions 500 bp upstream of TSSs, and another screening was done in the regions 1000 bp upstream of TSSs. Note that as *Dictyostelium* genome is poorly annotated, TSS positions often actually correspond to the start codon (for most protein-coding genes the locations of UTRs are not known). What is therefore considered a promoter may actually encompass a 5′ UTR region. This problem was manually curated for some selected genes, but could not be addressed for all transcripts. What is therefore labeled as ‘promoter’ in this manuscript may actually correspond to real promoter+5′ UTR. To obtain a rough estimate of the 5′ UTR length in *Dictyostelium*, we analyzed the upstream regions of a subset of 96 highly expressed genes for which there is high RNA-Seq and expressed sequence tags (EST) coverage. For these genes, the average 5′ UTR length appears to be lower than 100 bp (see Supplementary Methods and Figure S8). If we assume that this value holds true for most *Dictyostelium* genes, one can consider that upstream regions of 500 or 1000 bp may be correctly defined as promoter regions for most genes.

The mean of the scored nucleic acid sequence is computed for a sliding window length arbitrarily set at 25 nucleotides (for G4Hunter principle see the reference (25)). Enrichment for G4FS in genomic features was computed with an in-house script using GenomicFeatures package tools. All bioinformatics procedures are described in a R-script provided as a supplementary file.

Regarding G4Hunter analysis, we advocate the use of different thresholds depending on the application or target (RNA, DNA). Rather than giving a Yes/No answer, G4Hunter provides a propensity score. In other words, some sequences may be more or less G4-prone, and a number of parameters may facilitate or hamper G4 formation. For example, any sequence with a score above 2.0 is extremely likely to form a G4, and this quadruplex will be very stable. While we understand that statements like ‘Dicty contains XX G4-prone motifs’ are simple, we believe that they are misleading. For this reason, we systematically perform our analyses with two or more different thresholds.

### Selection of sequences

The first selection of candidates involved more than 14 sequences. However, the difficulty in determining the localization of the selected sequences relative to the genes (*e.g*. in promoter or UTR regions) due to the lack of annotation in the *Dictyostelium* genome incited us to restrict our study on a subset of motifs. We based our selection on two main criteria: (i) based on our experience with G4 formation *in vitro*, we selected sequences with a high probability of forming G-quadruplexes *in vitro* and (ii) we selected the sequences located in genes with important functions in *D. discoideum*.

### Oligonucleotides

Oligonucleotides were purchased from Eurogentec (Seraing, Belgium) and delivered in lyophilized form. Stock solutions were prepared in bi-distilled water. Concentrations were determined by absorbance spectroscopy at 260 nm using the Beer-Lambert law and the molar extinction coefficient provided by the manufacturer. The oligonucleotide stocks were stored at −20°C.

### Circular dichroism spectroscopy (CD)

Circular Dichroism experiments were carried out using a JASCO J810 spectrophotometer, thermostated by a Peltier. DNA samples concentrations and buffers used were the same as those considered for UV absorbance thermal denaturation experiments (4 μM). Three accumulations were performed and averaged between 220 and 335 nm at 25°C. The chosen acquisition parameters were 100 nm/min for the scanning speed with an interval of 1 nm between each reading and 1 sec for the response time. The measurements were performed in quartz cuvettes (Hellma, France) with 1 cm optical path. A baseline corresponding to the buffer was also recorded and was subtracted from the accumulation of spectra after exporting raw data. Topology was proposed according to (37).

### Absorbance spectroscopy

All experiments were performed in a 10 mM lithium cacodylate buffer at pH 7.2 containing 100 mM or 10 mM KCl, except for RNA sequences which were folded in 50 mM KCl. The G-quadruplex-forming oligonucleotides were typically prepared at 4 μM strand concentration.

Thermal denaturation and renaturation curves were obtained with SAFAS (Monaco) or Uvikon XL spectrophotometers using quartz optical cells of 1 cm path length (38,39) and a temperature gradient of 0.2°C/min. Even at this slow rate, some samples (p14 for example) exhibited significant hysteresis (heating and cooling profiles were not superimposed, complicating an accurate determination of *T*_m_). For these experiments, the usual salt concentration was 100 mM KCl, unless the sequence was so stable that we had to use a lower concentration (50 mM KCl for RNA sequences; 10 mM KCl for p172 which was extremely stable).

Thermal difference spectra (TDS) were obtained by taking the difference between the absorbance spectra of unfolded and folded oligonucleotides that were recorded at high (94°C) and low (4°C) temperatures, respectively, in a potassium-containing buffer.

Isothermal difference spectra (IDS) were obtained as described previously by taking the difference between the absorbance spectra from unfolded and folded oligonucleotides in presence or in absence of KCl at 25°C (40). For the interpretation of the TDS and IDS spectra shape, see reference (25).

### Nuclear magnetic resonance (NMR)

One-dimensional (1D) 1H nuclear magnetic resonance (NMR) experiments were performed on a Bruker Advance 400 MHz instrument. 1D 1H NMR experiments were performed with a ‘jump-return’ water suppression and a number of scans between 1024 and 2048 depending on the sample concentration. For all the sequences, NMR spectra were recorded at 298 K. The samples were first denatured at 90°C in water before adding 20 mM potassium phosphate buffer (KPi) pH 6.9, 70 mM potassium chloride (KCl) and 10% of deuterated water (D_2_O). The samples, at a strand concentration of around 100 μM, were again heated at 90°C before allowing to cool overnight.

### Gel electrophoresis

Non-denaturing polyacrylamide gel (15%) electrophoresis was used to visualize the oligonucleotides. The gel was supplemented by 10 mM KCl. DNA and RNA samples were already folded for NMR (see conditions above) at 100 μM, and were diluted at 30 μM. A concentration of 100 μM corresponds to the NMR conditions while 30 μM was the minimum DNA/RNA concentration we could easily visualize by UV shadow. Sucrose was added to the samples at a final concentration of 23%. Oligothymidylate markers (dT_21_, dT_15_ and dT_9_) were loaded in parallel on the gel. Electrophoresis was performed at 4 W per gel to reach a temperature close to 19°C. After electrophoresis, the gels were visualized by UV shadowing with MF-ChemiBIS 3.2 bioimaging system, then they were stained by Stains-All solution (Sigma-Aldrich) for 10 min under gentle agitation. After that, they were incubated in water for discoloration under light before visualization on the same system.

### *D. discoideum* strains and cell culture

*Dictyostelium discoideum* strain HMX44A was provided by Dr. Pierre Golstein from the immunology center of Marseille-Luminy (CIML). HMX44A cells were grown in HL-5 axenic media (Formedium) based mainly on peptone and yeast extract. The strains were cultured at 22°C on plastic dishes. AuMA is a porphyrin derivative developed at LCC, Toulouse (41,42). AuMA was dissolved in water at a final concentration of 6 mM and the solutions were stocked at 4°C.

### RNA isolation and qRT-PCR

HMX44A cells were seeded in 6 cm Petri dishes and treated with different concentrations of ligand (10, 20 and 30 μM). After 24 h, the cells were lyzed and the total RNA was extracted according to the instructions provided by the manufacturer (RNeasy, Qiagen). To remove possible genomic DNA contamination, the isolated RNA was treated by DNase I. One μg of RNA was reverse transcribed by using the enzyme M-MLV RT and all the reagents were purchased from Promega. The cDNA was quantified using the kit Brilliant II SYBR^®^ Green QPCR Master Mix from Agilent technologies and the thermocycler Stratagene Mx3005P was used to carry out the real time PCR experiments. Histone H3a gene (DDB0191157 / DDB_G0267402) was used as an internal control for normalization. The forward and reverse primer sequences used for amplification were:

- for H3a (F) 5′ GTACCACGTAAACACATTGG 3′ (R) 5′ CCTGGTCTGAAACGATGTA 3′,
- for p10 (F) 5′ CATCCTGGTTTCGCTGTA 3′ (R) 5′ CTAAAGCACTACCCATATCA 3′,
- for p32 (F) 5′ GTATCACCTTAACCTCAAGAAAC 3′ (R) 5′ GACCCTTAGCCTTGACTTT 3′,
- for p187 (F) 5′ GCACTGACAATGATCCATAAA 3′ (R) 5′ TAGCAGACGATTCACCAC 3′,
- for p193 (F) 5′ CATTGCATATGGCACTCAAA 3′ (R) 5′ ATCGGTCTCTTGTATTTGACC 3′ and
- for p40 (F) 5′ TCGGTCAATGGAAACATTG 3′ (R) 5′ GACCTGATCCAATGTATTTAGCTAA 3′.

### FRET melting

A Stratagene Mx3005P instrument was used to carry out the FRET melting experiments in 96-well plates as previously described (43). After an initial incubation at 25°C for 5 min, the temperature was increased by 1°C every minute until 95°C was reached. Each experimental condition was tested in duplicate. The mid-transition *T*_1/2_ was determined from normalized curves. The experiments were performed with samples containing 0.2 μM of F21T, 10 mM lithium cacodylate (pH 7.2), and 10 mM concentrations of KCl with 90 mM LiCl. F21T is the human telomeric G4 sequence with the fluorescent dyes FAM and TAMRA attached to the 5′ and 3′ ends, respectively (FAM-(GGGTTA)_3_GGG-TAMRA). The *Dictyostelium* sequences p10, p187, p193, p40 and p32 were used in large excess at 3 and 10 μM. For measurements in the presence of a ligand, the concentration of AuMA was 0.5 μM. The stabilization induced by a ligand is reported in terms of the difference in the mid-transition temperature with and without the ligand (ΔT_1/2_).

## RESULTS AND DISCUSSION

### Bioinformatics mapping of G-rich sequences in *D. discoideum* by G4Hunter

While hundreds of thousands of putative G4-forming sequences (G4FS) have been predicted in the human genome, far less G4FS are expected to be found in *D. discoideum* promoters, exons and introns. Applying G4Hunter to the genome of *D. discoideum* (strain AX4 (34)) we found 249 (threshold = 2.0) to 1055 (threshold = 1.5) G4-prone motifs, as expected for a small size and AT rich (78%) genome (Table 1). The calculated density of G4-prone motifs (number of hits per kb) is 0.007 and 0.031, for threshold values of 2.0 and 1.5 (Table 1), respectively. These densities are 16 and 17-fold lower than the corresponding densities in the human genome using identical thresholds. In other words, given that the human genome is >100 times larger, it has over 1000 times more G4-prone motifs than *Dictyostelium*.

**Table 1.** Number, density and relative density of G4 motifs found in *D. discoideum* genome with G4Hunter. For each genomic feature, we report the number of G4 motifs that overlap the feature coordinates. We did not take into account the strand information for the feature and the G4

	1.5			1.75			2		
G4Hunter Threshold	n	density^a^	relative^b^	n	density	relative	n	density	relative
Prom500^c^	349	0.044	*1.42*	160	0.020	*1.50*	85	0.011	*1.47*
Prom1kb^c^	612	0.043	*1.40*	274	0.020	*1.48*	154	0.011	*1.54*
Trans	582	0.024	0.76	237	0.010	0.73	122	0.005	0.67
CDS	328	0.016	0.52	102	0.005	0.37	41	0.002	0.28
Exons	464	0.020	0.64	165	0.007	0.52	68	0.003	0.39
Introns	157	0.063	*2.03*	94	0.037	*2.77*	62	0.025	*3.35*
intergene	490	0.056	*1.79*	229	0.026	*1.92*	131	0.015	*2.03*
**Whole genome** ^d^	**1055**	**0.031**	1.00	**459**	**0.014**	1.00	**249**	**0.007**	1.00

^a^Number of hits per kb: number of hits in the left column divided by the length of the feature.

^b^Relative density as compared to density for the whole *D. discoideum* genome = 1.00. Higher than average densities are shown with italic digits.

^c^See comment on promoter definition in the Materials and Methods section; these regions may also include 5′ UTR regions.

^d^Total number is not the sum of categories above as a G4 sequence may be listed in several categories.

We compared the relationship between the global G4FS density and the threshold used for G4Hunter between *D. discoideum* and the other organisms we analyzed in the original G4Hunter article (25). For most organisms, this relationship can be fitted with a single exponential law but 2 regimes were necessary to describe the relationship for *D. discoideum* (Supplementary Figure S1A). At high threshold (>1.4); the slope (–3) is similar to the one observed for the human genome (and most of the organisms we reported), but for low thresholds (≤1.4), the slope is steeper (–4.5) and similar to the one for the unicellular organisms we reported (*E. coli, S. pombe, S. cerevisiae*). This could be related to a tendency unicellular organism to avoid too many G4-prone sequences, thus the steep decrease for low thresholds. The relative conservation of G4FS at higher thresholds could result from the need to keep some G-rich motifs for binding of specific regulatory factors with an important biological function.

We then investigated whether G4 density depended on genomic location. At all tested thresholds the G4FS density appeared to be slightly higher on chromosome 6 (Supplementary Figure S1b) with *P*-values ranging from 0.03 (threshold = 1.2, 1.5) to 0.07 (threshold = 1.75). We did not find any similar bias for the gene-related features we tested (promoters, transcripts, CDS, exons, introns).

Despite the overall paucity in G4-prone motifs, there are 23 sequences in the genome of *D. discoideum* which contains multiple runs of at least 4 guanines (Supplementary Table S1a). Most of them (12/23) are short (*n* ≤ 6) repeats of G rich motifs: (GGGGACA)_n_, (GGGGA)_n_, (GGGGTT)_n_, (GGGGGAA)_n_ and (GGGGTTGTT)_n_; or oligo G repeats (G_19–22_) (in italics). Among the remaining 11 sequences, three have only two very long track of G’s making them hard to study by biophysical approaches. The three-dimensional structure of one of these quadruplexes has been recently solved (40). Interestingly, a low GC% does not prevent the rare occurrence of long G4-prone motifs: the five longest sequences found with G4Hunter with a threshold of 2 are shown in Supplementary Table S1b; one motif is over 100 nucleotide-long.

**Figure 1.**
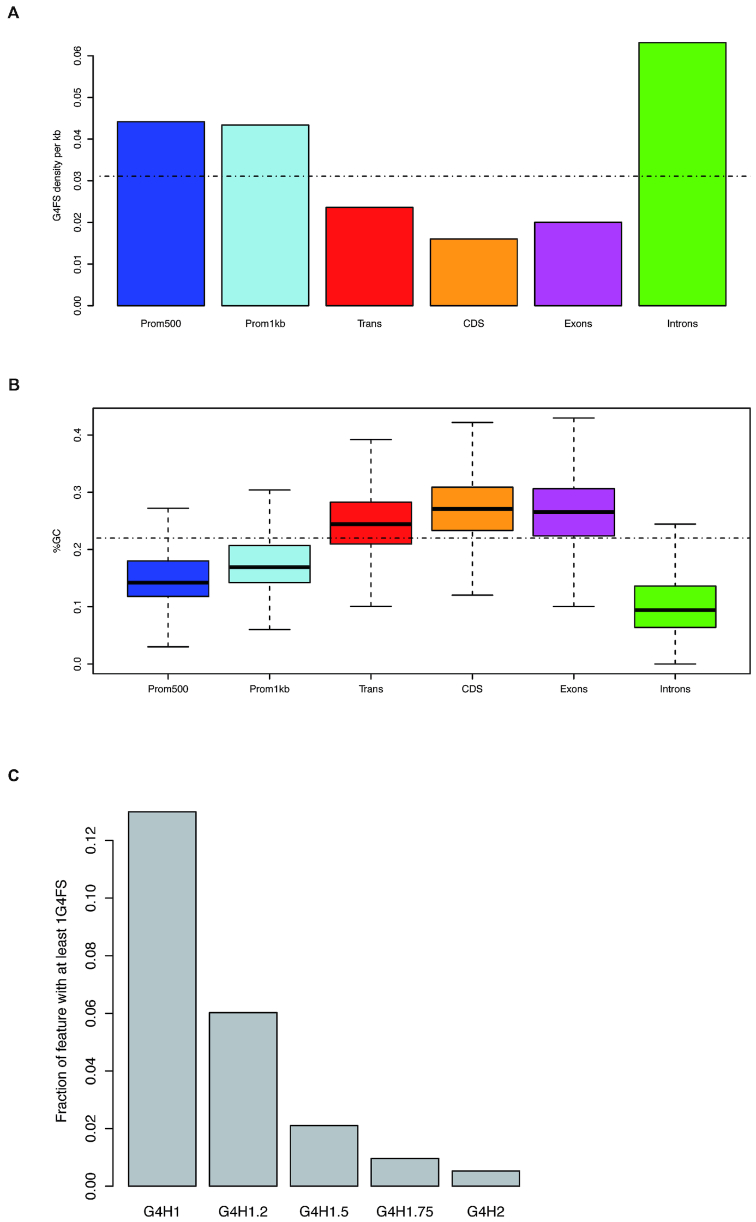
(**A**) The density of G4 motifs (G4FS) was calculated by dividing the number of G4FS found with G4Hunter (threshold = 1.5) overlapping the given genomic feature by the total length of the feature in kilobase. The dotted line indicates the average G4FS density for the whole genome. Prom500 and Prom1kb indicate promoters defined as 500 or 1000 bp upstream of genes, respectively. (**B**) Distribution of the GC content for the different features represented as a boxplot (computed with default parameters in R, v3.5.1). The dotted line indicates the average GC content of the *D. discoideum* genome. (**C**) Fraction of promoters (defined as 500 bp upstream of genes) with at least one G4FS for different thresholds. This fraction was calculated by counting the number of promoters overlapping with at least one G4FS at a given threshold divided by the number of promoter regions.

Genome annotation for *D. discoideum* is less complete than for other model organisms; we could nevertheless analyze G4 density in various regions of the genome (Table 1 and Figure 1A). A striking and counterintuitive feature is that G4 enrichment is not correlated with the relative GC content: ‘promoter’ regions and introns exhibit higher-than-average G4 densities and have a lower-than-average GC content (Figure 1B). This illustrates that GC% is not the most important parameter influencing G4 density: G-clustering and GC skewness are key contributors to G4 propensity.

As UTRs are not annotated in the *D. discoideum* genome, the promoters were derived from the genome annotation downloaded (http://dictybase.org/). The promoter G4 screening was done using different arbitrary definitions of promoter regions. Core promoters were defined as the 500 nucleotides upstream of the indicated ‘TSS’ (which actually corresponds to the start codon of CDS) [–500, +1]; and a more relaxed definition included 1000 nucleotides upstream of the TSS/CDS [-1000, +1]. Using a subset of ∼100 highly expressed genes for which there is high sequence coverage, the length of the 5′ UTRs was estimated to be usually shorter than 200 bp (with an average length <100 bp; see Supplementary Methods andSupplementary Figure S8): these promoter arbitrary definitions can therefore be considered as correct for most genes. In particular, ≈2% of core promoters contain at least one G4 promoter sequence with a score of 1.5 or higher. This proportion drops below 1% for 1.75 or higher (Figure 1C). The real fraction of *bona fide* promoters with a G4 motif could even be lower, due to the lack of UTR annotation: some G4 motifs counted in the promoters may actually be located in the 5′ UTR region.

In addition to promoter regions, we also found an enrichment of G4-prone sequences in intronic regions (Supplementary Figure S2A and B). As for promoters, this enrichment is still relative, as only 157 or 62 introns (out of a total of 18,192; *i.e*. <1%) contain a G4 motif with a threshold of 1.5 or 2.0, respectively. However, this enrichment is unlikely to be relevant at the RNA level, as it is only found on the *non-coding* strand (i.e. corresponding to C-rich regions in the mRNA intron). Interestingly, this is in complete opposition to what was found in mammals, with an over-representation of G-rich motifs in the first introns of human genes (12,25). This strand bias for G4-prone motifs is not related to a trivial bias in G content (Supplementary Figure S2C).

### Selected genes with putative G4-forming sequences in *D. discoideum*

Among the G4 hits found in *D. discoideum* genes, we selected 14 sequences with high probability to form G-quadruplex based on our experience on G4 formation *in vitro*. According to the data we have in hands from the EST alignments and RNA sequencing in dictyBase (http://dictybase.org/), these sequences are located in gene promoters (and possibly in 5′ untranslated regions), in exons or are suspected to form in non-coding RNA.

Interestingly, several of these genes play potentially important roles in *D. discoideum* (Table 2). Sequence **p172** is located in the Ras gene promoter (*rsmJ*), while **p40** is located in the promoter of the gene coding for phosphatidylethanolamine-binding protein PEBP. PEBP is a multifunctional molecule regulating several important cellular signal pathways. **p16** is located in the promoter of (*tgrR2*) coding for immunoglobulin E-set domain-containing protein. **p32** is located in the promoter of (*rps20*) coding for 40S ribosomal protein S20. These sequences are located 359, 224, 183 and 75 bp upstream of the start codon, respectively. **p220** and **p309** are located in gene exons. The former is located in exon 4 of DDB_G0288639 that codes for a conserved heat shock protein from DnaJ homolog subfamily C member 7. The latter is located in exon 2 of *dyd*A gene coding for a Ras effector protein playing important role in chemotaxis in *D. discoideum*. p23 is located probably in the 5′ UTR region of DDB_G0349518.

**Table 2.** Selected sequences in *D. discoideum* genome. The selected G4-prone sequences with their name, function, length, probable distance from annotated ORF and the dictyBase Gene ID

Sequence name	Gene function and name	Gene ID	G4 sequence (5′ = > 3′)	Genomic location	DNA	RNA	Oligo. length (nt)	Distance to TSS (bp) (n)
**p16**	Immunoglobulin E-set domain-containing protein (*tgrR2*)	DDB_G0277627	AGGAGAGTGGGGGTGTGTGTTTGGGTGGGT	Chr2, 8277511–8277540	x		30	-183
**p32**	40S ribosomal protein S20 (*rps20*)	DDB_G0278429	AGGATTGGGGCTGGGTGTGCGGGA	Chr3, 872263-872240	x		24	-75
**p40**	Phosphatidylethanolamine-binding protein PEBP	DDB_G0283803	TGGGGGGAAGAGGGGAGAGACAGGGGGAGGTAGG	Chr4, 1122650–1122683	x		34	-224
**p172**	Small GTPase (*rsmJ*)	DDB_G0279305	GGGGGTGGGGGTGGGGTAAGGGG	Chr3, 1907066–1907088	x		23	-359
**p23R**	Unknown	DDB_G0349518	UGGGAUGGGGUGACAGUUGGGGGGAGAGGGGUAUGAUG	Chr2, 5616940-5616903		x	38	(a)
**p187**	Similar to serologically defined breast cancer antigen 84 (sdbcag84/ERGIC3)	DDB_G0280993	ATGGGAGGTAGATGGTGGGTGGGTGGTGA	Chr3, 3920891–3920919	x	x	29	(b)
**p193**	Cell migration and cell-cell adhesion (*sma*)	DDB_G0281803	AGGGGATGGTTAGCGGTGCGACAGGGGGGAGGAGACAGGGGGA	Chr3, 4732632–4732674	x	x	43	(c)
**p4**	Hypothetical protein		AGGTGGGGGTGAGCAGTGTGGTGTGGGT	Chr1, 717709-717682	x	x	28	(d)
**p12**	Hypothetical protein	DDB_G0276613	TGGTGGTGGTAGGAAGTGGT	Chr2, 7074056–7074075	x	x	20	(e)
**p14**	Hypothetical protein	DDB_G0276707	TGGTGGAGGGGGGTGGGTGGT	Chr2, 7077389–7077409	x	x	21	(f)
**p5**	Hypothetical protein		TGGGGTTGGATGGGTGGGT	Chr1, 1755973-1755955 Chr1, 1756455-1756437	x		19	(g)
**p220**	Conserved protein, DNAJ heat shock N-terminal domain-containing protein	DDB_G0288639	TGGGAGGTGGTATGGGAGGTGGAGGC	Chr5, 1758405–1758430	x	x	26	(h)
**p309**	Putative Ras effector, chemotaxis (*dydA*)	DDB_G0287875	TGGGGGGAGGTGGTGGTGGTGGTGGAT	Chr5, 631724–631750	x	x	27	(i)
**p10**	Short-chain dehydrogenase*/*reductase (SDR) family protein	DDB_G0295833/ DDB_G0273855	GGGGGAGGGGTACAGGGGTACAGGGG	Chr2, 2545914-2545889 Chr2, 3485872–3485897	x		26	(j)

(n) The localization of G4 sequence is determined based on the data from RNA-Seq and EST alignments available in dictyBase.

(a) G4 is localized in 5′ UTR of DDB_G0349518.

(b) G4 is localized in the promoter of DDB_G0280993 (similar to serologically defined breast cancer antigen 84) or in noncoding RNA gene DDB_G394564.

(c) G4 is localized in the promoter of DDB_G0281803 (*sma*, which plays a role in cell migration and cell-cell adhesion) or in 3′ UTR of DDB_G0281631 (pseudogene).

(d) G4 is localized in 5′ UTR or promoter of DDB_G026833 or within upstream ncRNA gene DDB_G3967196.

(e) G4 is localized in promoter of DDB_G0276613.

(f) G4 is localized in promoter of DDB_G0276707.

(g) Not enough coverage to determine the exact localization (UTR or promoter). There are two copies of p5 flanking the gene DDB_G0268466 (∼100 bp upstream and downstream). In addition, three and five copies of p5 interspersed by ∼400 bp are found in intergenic regions in chromosome 2 (4788017-4788460) and 5 (849448-850835), respectively.

(h) Located in exon 4 of DDB_G0288639 that codes for a conserved heat shock protein from DnaJ homolog subfamily C member 7.

(i) Located in exon 2 of *dyd*A gene (DDB_G0287875) coding for a Ras effector protein playing an important role in chemotaxis in *D. discoideum*.

(j) **p10** can be mapped at 263 and 86 nucleotides upstream of two divergent genes (DDB_G0295833*/*DDB_G0273855 and DDB_G0273115*/*DDB_G0273853), respectively. Gene ID DDB_G0295833*/*DDB_G0273855 is annotated as a short-chain dehydrogenase*/*reductase (SDR) family protein whereas no functional annotation is available for Gene ID DDB_G0273115*/*DDB_G0273853. It is likely to be in the promoter region due to proximity to the genes (33). There are two copies of **p10** and its neighboring genes as they are part of a 750-kb region that is duplicated in chromosome 2 of strain AX4.

For the six remaining sequences, the information provided by dictyBase on RNA sequencing and EST alignment is insufficient to conclude on their exact location. Some of these G4 may have important functions. **p187** may locate in the promoter of a gene coding for DUF1692 family protein, similar to serologically defined breast cancer antigen 84 (sdbcag84/ERGIC3). In addition, **p193** may locate in the promoter of a gene playing a role in cell migration and cell-cell adhesion in *D. discoideum*. Finally, **p10** is a unique sequence forming a very stable quadruplex. This sequence can be mapped at 263 and 86 nucleotides upstream of two divergent genes. One of them is annotated as a short-chain dehydrogenase*/*reductase (SDR) family protein whereas no functional annotation is available for the other one. **p10** is likely to be in the promoter region due to the proximity to the genes. Importantly, we determined the crystal structure of this quadruplex in a recent work (40). The information about the localization of all these G4FS is presented in Table 2.

### Characterization of G4 formation by different spectroscopic methods

We determined the ability *in vitro* of these 14 selected sequences (DNA and RNA oligonucleotides) to form G-quadruplexes with standard spectroscopic assays UV melting, circular dichroism, UV thermal difference spectra (TDS) and UV isothermal difference spectra (IDS). Salt concentrations were chosen to mimic eukaryotic physiological conditions.

**Figure 2.**
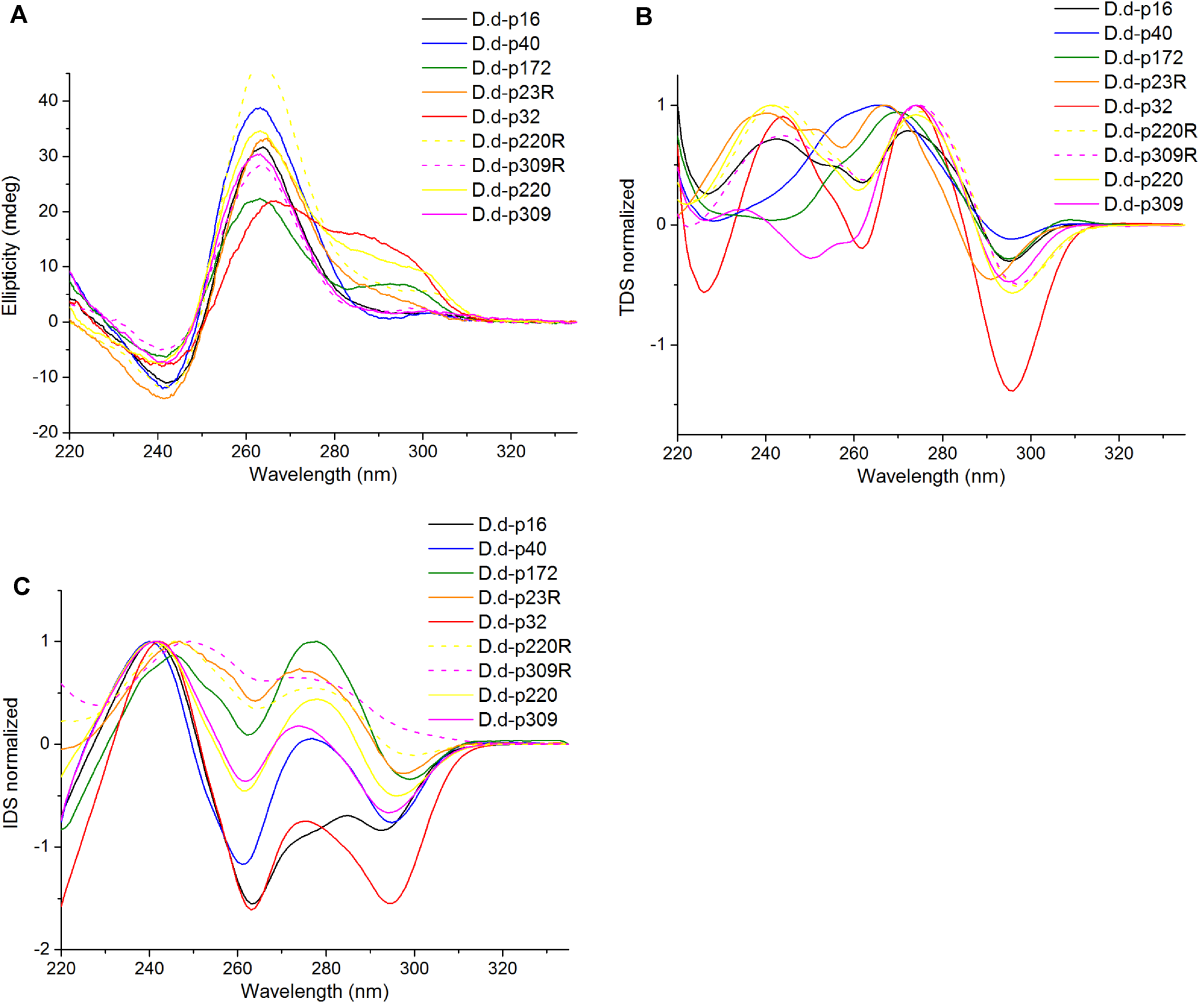
Characterization of **p16, p32, p40, p172, p23R, p220, p309, p220R** and **p309R** by different biophysical methods. (**A**) CD spectra done at 25°C; (**B**) thermal difference spectra (TDS); (**C**) isothermal difference spectra (IDS) at 25°C. The DNA samples were folded in 100 mM KCl at 4 μM except **p172** which was folded in 10 mM KCl and the RNA sequences were folded in 50 mM KCl. TDS and IDS spectra were normalized to [0;1].

Circular dichroism (CD) was carried out at first under potassium buffer conditions in 100 mM KCl to determine the G-quadruplex conformation (10 mM KCl for p172; all the RNA sequences were folded in 50 mM KCl). All the tested sequences presented CD signals indicative of G4 formation. **p16** and **p40** (located in promoters) as well as the **p23R** RNA sequence showed a positive peak at 263 nm and a negative one at 242 nm characteristic of parallel G4 conformation (37). Both the DNA and RNA sequences of **p220** and **p309** (located in exons) presented a parallel conformation. Similarly, **p187, p193, p14**, their corresponding RNA sequences and **p5** exhibited a parallel conformation. In contrast, the CD spectra of **p32** and **p172** present in the *Ras* gene presented a large positive peak at 293nm, a well-defined peak at 263 nm and a negative peak at 243 nm characteristic of a mixed conformation (Figure 2A). Finally, **p4, p12** and **p10** presented antiparallel conformations with positive peaks at 293 nm and negative peaks at 263 nm (37), however, **p4R** and **p12R** (the corresponding RNA) presented a parallel conformation (Supplementary Figure S3A). These observations are summarized in Table 3.

**Table 3.** Melting temperatures of the different G4 sequences calculated according to UV absorbance melting profiles recorded at 295 nm, and their G4 conformation concluded from CD signature. R indicates the corresponding RNA sequence. *T*_m_ determinations were calculated based on the heating curves and repeated at least twice (*T*_m_ values were reproducible within 2–3°C)

Sequence name	Probable G4 conformation^a^	*T*_m_ (°C)	G4Hunter score
**p16**	Parallel	56^b^	1.57
**p32**	Mixed	51^b^	1.58
**p40**	Parallel	57^b^	2.06
**p172**	Mixed	77^c^	3.13
**p23R**	Parallel	67^d^	1.82
**p187**	Parallel	61^b^	1.41
**p187R**	Parallel	78^d^	1.41
**p193**	Parallel	56^b^	1.67
**p193R**	Parallel	61^d^	1.67
**p4**	Antiparallel	42^b^	1.46
**p4R**	Parallel	60^d^	1.46
**p12**	Antiparallel	36^b^	1.05
**p12R**	Parallel	45^d^	1.05
**p14**	Parallel	52^b^	2.14
**p14R**	Parallel	72^d^	2.14
**p5**	Parallel	55^b^	2.00
**p220**	Parallel	68^b^	1.42
**p220R**	Parallel	65^b^	1.42
**p309**	Parallel	60^b^	1.78
**p309R**	Parallel	78^b^	1.78
**p10** ^e^	Antiparallel	>90^b^	2.54

^a^As inferred from CD shape.

^b^Determined in 100 mM KCl.

^c^Determined in 10 mM KCl.

^d^Determined in 50 mM KCl.

^e^See (40).

**Figure 3.**
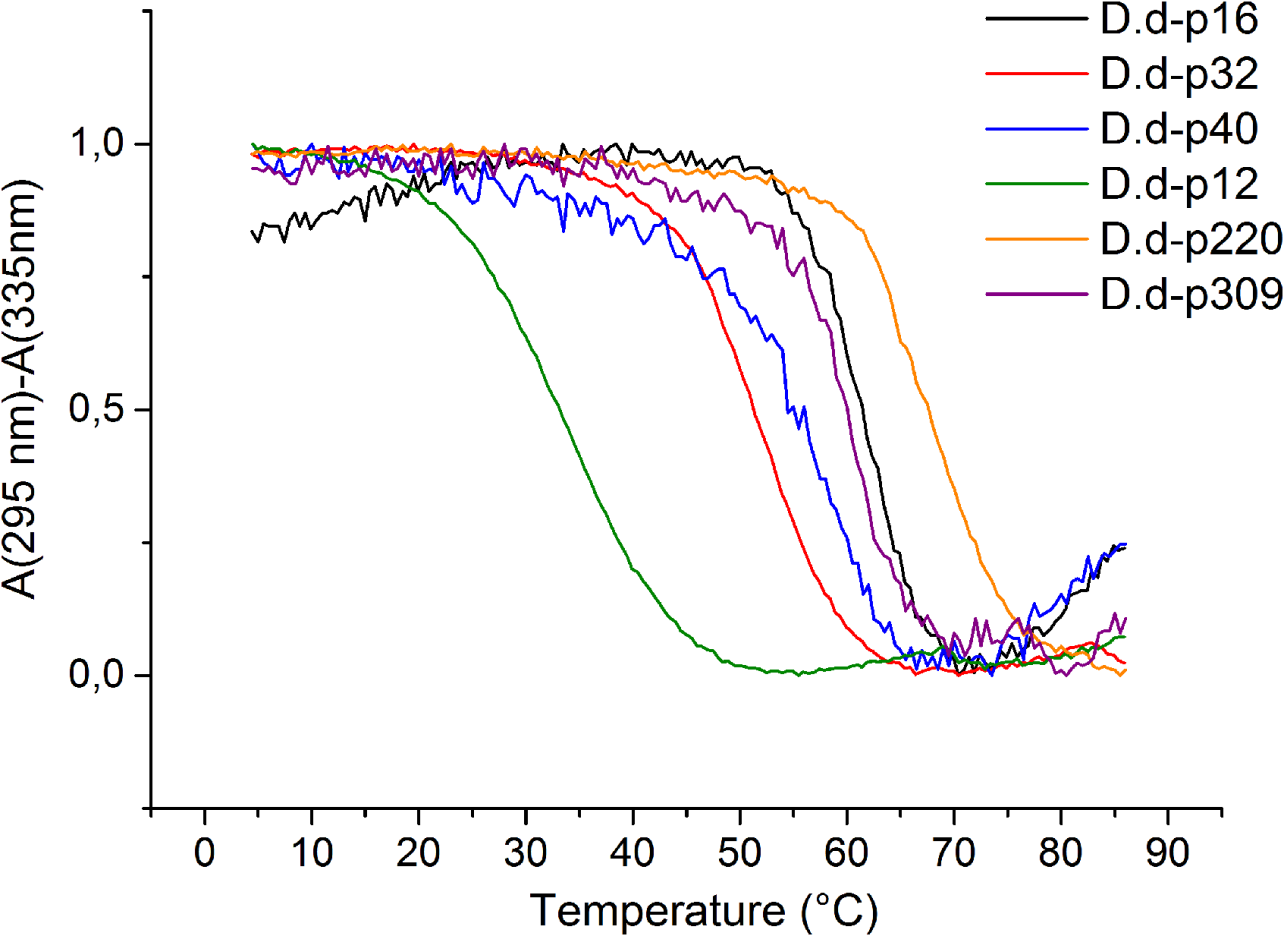
UV melting and annealing curves recorded at 295 nm after baseline substraction recorded at 335 nm. The DNA samples were folded in 100 mM KCl at 4 μM.

To confirm G4 formation, UV melting experiments at 295 nm were carried out for all sequences. All melting curves presented an inverted sigmoid shape as expected for G-quadruplexes (38,39). The cooling and heating curves of **p32, p172, p220**, p**309** and **p12** were nearly superimposable, suggesting intramolecular folding (Figure 3; Supplementary Figure S4). All DNA sequences were studied in 100 mM KCl (except **p172** which was folded in 10 mM KCl) and all the RNA sequences were folded in 50 mM KCl given their high stability. Melting temperatures are provided in Table 3.

As a part of the spectroscopic methods as well, to confirm the G-quadruplex formation, UV thermal difference spectra (TDS) were recorded on these sequences by subtracting the absorbance spectrum at 4°C (where the G4 is fully folded) from the absorbance spectrum at 94°C (where the G4 is generally fully unfolded). Major negative peaks at 295 nm with positive peaks at 273 nm were observed. These peaks are typical for G-quadruplex formation. Among these sequences, some of them presented a less intense peak at 295 nm, these sequences have a lower propensity to form G4 or form heat-resistant G-quadruplexes (**p40, p172, p193, p193R, p4, p14, p14R** and **p309)** (Figure 2B; Supplementary Figure S3B).

In addition, Isothermal difference spectra (IDS) were recorded at 25°C by subtracting the absorbance spectra obtained after adding Potassium buffer (folded form) from the absorbance spectra obtained before adding Potassium buffer (unfolded form). The IDS spectra showed negative peaks at 295 nm and positive peaks at 273 nm that are characteristic of G4 formation. As for TDS, **p187, p193R, p4R, p14R** and **p220R** presented a less intense peak at 295 nm. Only **p309R** did not present a defined negative peak at 295 nm. (Figure 2C; Supplementary Figure S3C).

**Figure 4.**
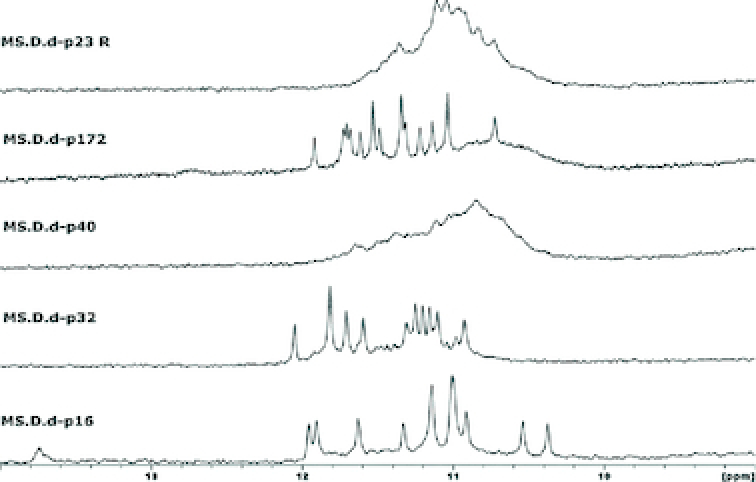
1D Imino proton spectra in 20 mM potassium phosphate buffer at pH 6.9 with 70 mM KCl at 25°C.

In addition, for further confirmation, proton NMR experiments were done at 25°C. Although some spectra were poorly defined, all of them exhibited the characteristic imino peaks in the 10−12 ppm range. The most stable sequence **p172** (*Ras* gene) displayed well-defined 16 imino peaks corresponding to four G-quartets (Figure 4; Supplementary Figure S5).

Altogether, these biophysical results demonstrate that all candidate sequences do form stable G-quadruplexes *in vitro* under physiological conditions, highlighting G4Hunter accuracy.

### Determination of molecularity by non-denaturing PAGE

We then aimed at determining the molecularity of the selected *D. discoideum* sequences by gel electrophoresis. Sequences were already prepared and folded in 20 mM potassium phosphate buffer (KPi) at pH 6.9 containing 70 mM potassium chloride (KCl) and 10% of deuterated water (D_2_O) for NMR. The same samples were used for gel electrophoresis. The oligonucleotides were loaded at two different concentrations (100 and 30 μM) on native polyacrylamide gels supplemented with 10 mM KCl. Single stranded oligothymidylate dT_n_ oligonucleotides were used for comparison. The bands were revealed first by UV shadowing (data not shown) and then with ‘Stains-All’ staining (Supplementary Figure S6). The intensities of the bands correlate with the concentration of the oligonucleotide used. The gels revealed that **p12** and its corresponding RNA sequence **p12R** form one band at 100 and 30 μM suggesting a unimolecular folding form in solution. This result was confirmed by UV melting data (Supplementary Figure S4), as the absence of hysteresis between the cooling and denaturation curves suggests that these sequences form intramolecular G-quadruplexes. For all the other sequences, the presence of multiple bands suggests the folding into different G4 forms in solution. The different molecularities of these complexes could not be determined precisely and additional structural studies are needed to fully understand their folding.

### AuMA, a G4-specific ligand, stabilizes *Dictyostelium* G4 sequences *in vitro*

After confirming G-quadruplex formation *in vitro* for the selected 14 sequences, we investigated the biological effects of G4 ligands on the expression of *D. discoideum* genes containing a G4 motif in their upstream promoter region. Among the 14 sequences, five are likely (based on EST and RNA-Seq data from dictyBase) located in promoter regions: **p32, p40, p10, p187** and **p193**. The G4 motif is in the immediate vicinity of the start codon for **p32** and **p40** (75 and 224 bp upstream, respectively). However, for **p187, p193** and **p10** the data present on dictyBase was not sufficient to determine their exact location (Table 2).

**Figure 5.**
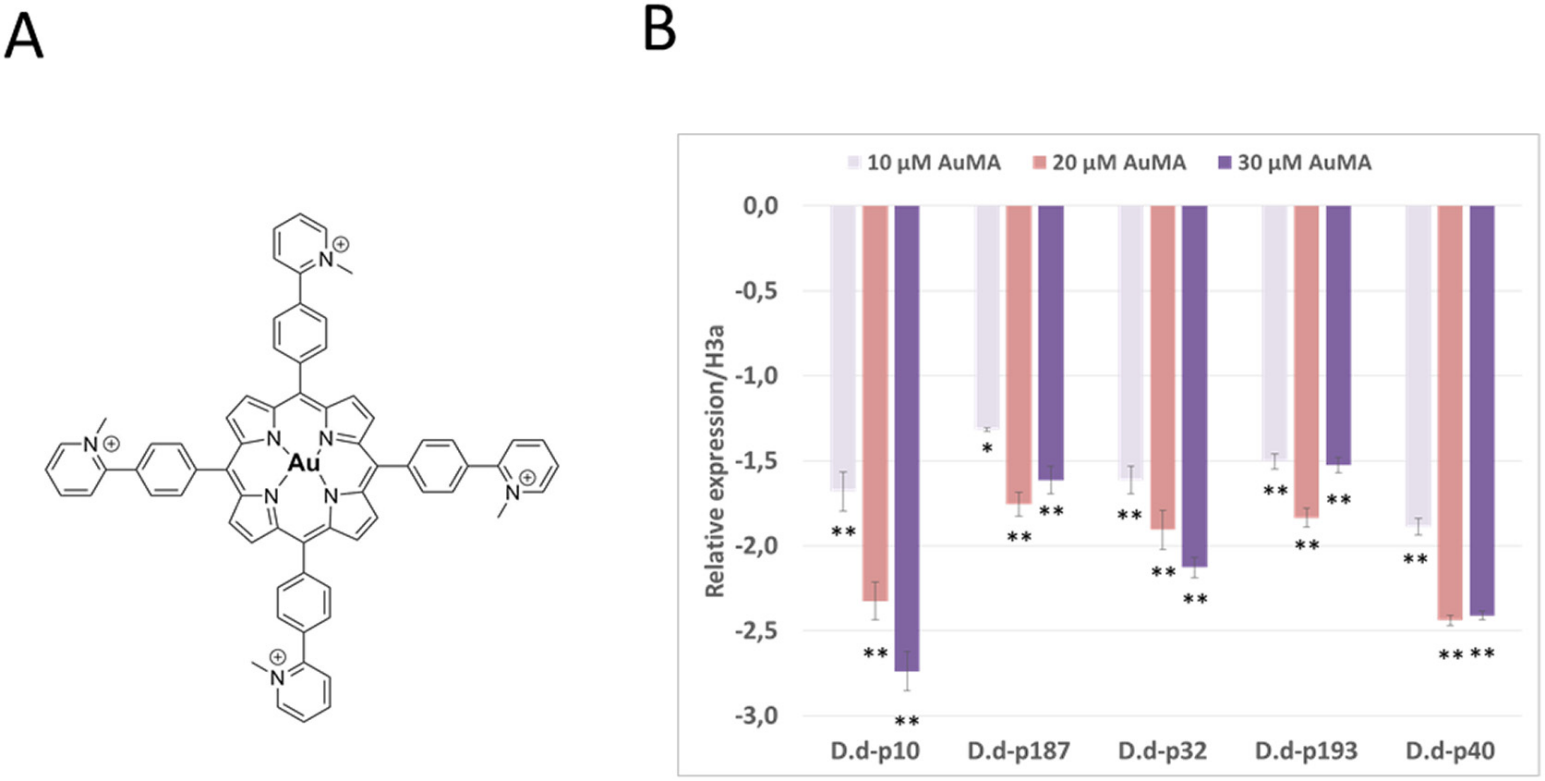
(**A**) Structure of AuMA, a G4 ligand porphyrin derivative. (**B**) AuMA inhibits the transcription of G4-prone *D. discoideum* genes. *D. discoideum* HMX44A cells were treated with increasing concentrations of AuMA (10, 20 and 30 μM) for 24 h and then the transcript levels of genes carrying **p10, p187, p32, p193** and **p40** G4 sequences in their upstream regions were measured by using real time qRT-PCR. The histograms show the relative fold change related to the internal control H3a. The control group is the condition without ligand treatment, and the corresponding variation in relative expression is zero. The data are presented as mean ± SD from two replicate wells and two independent experiments. The data were analyzed on GraphPad Prism and the paired Student's t-test (repeated measures one-way ANOVA) was applied. Asterisks indicate significant differences between treated cells and the control group. (*): (0.001 < *P*-value < 0.05); (**): (*P*-value < 0.001).

We chose a porphyrin derivative, AuMA, which is a high affinity G4 ligand and studied its effect on the expression of the five corresponding genes (Figure 5A). This compound has demonstrated antiproliferative and anti-HIV inhibitory effect via G-quadruplex binding (41,42).

Before studying the biological effect of AuMA, we assessed its stabilization effect on the five selected sequences by FRET melting assays. Without competitor, AuMA stabilized highly F21T (human telomeric G4 sequence labeled with FAM and TAMRA fluorescent dyes; Δ*T*_1/2_ ≈ 30°C). In the presence of a large excess of *Dictyostelium* sequences **p10, p187, p193, p32** and **p40** used at 3 and 10 μM, the Δ*T*_1/2_ was decreased greatly (by ∼25°C) demonstrating that AuMA stabilized also the *Dictyostelium* G4 sequences (Supplementary Figure S7).

### AuMA affects the expression of *D. discoideum* genes containing G4-prone motifs in their promoters

We then investigated the ability of AuMA to bind and affect the transcription of the selected *D. discoideum* genes. The effect of AuMA was tested using real time qRT-PCR. The *D. discoideum* strain HMX44A was treated by three different concentrations (10, 20 and 30 μM) of AuMA and the total RNA was isolated after 24h of treatment. The IC50 (inhibitory concentration) of AuMA in HMX44A was 30 μM after 24 h of treatment (data not shown). The expression of histone H3a gene served as a control. We already verified that these selected sequences were present in the HMX44A strain by PCR amplification followed by sequencing (data not shown).

AuMA decreased the expression of the different genes. The effect seemed low at 10 μM, however, at 30 μM, the effect of AuMA increased. The gene harboring **p10** was the most affected gene with ≈2.7-fold change at 30 μM AuMA, followed by genes harboring **p40** and **p32** (PEBP and *rps20*, respectively; Table 2). Genes with **p193** and **p187** (DDB_G0281803 and DDB_G0280993, respectively) were less affected, with fold changes of ≈1.5 (Figure 5B). Interestingly, the G4 sequence of **p10** is highly stable under physiological conditions and we recently solved its structure (40). These findings suggest that the five studied sequences are able to form G4 structures *in cellulo* and play a role in the expression of their target genes.

## CONCLUSION

The expected scarcity of G4-prone sequences in *D. discoideum* was confirmed using G4Hunter. The low number of candidate sequences (a few hundreds, as compared to hundreds of thousands in the human genome) should allow a detailed and nearly exhaustive examination of such motifs, at least *in vitro*. G4 densities vary depending on genomic location. Interestingly, (i) G4 density did not correlate with GC content: above-average densities were found in promoters and introns, which exhibit a GC content even lower than the average of the whole *Dictyostelium* genome; (ii) a significant enrichment was found in introns, as already observed for other species, but with a pronounced reversed strand preference, making extremely unlikely an involvement of RNA G4 formation in splicing in *Dictyostelium* (these introns would bear C-rich motifs; G4 formation would be possible on the complementary strand).

We performed an *in vitro* biophysical study of 14 sequences found in *Dictyostelium* promoters or transcribed regions. All sequences predicted by G4Hunter and tested here were shown using a battery of different techniques to adopt a quadruplex conformation. This reinforces our confidence in the accuracy of this algorithm. Interestingly, 11 of the 14 sequences escaped Quadparser consensus using default parameters (runs of 3+ G's separated by 1–7 nt loops), confirming that Quadparser easily misses *bona fide* G4-forming motifs and generates a number of false negatives. In addition, we demonstrated that the expression of five *D. discoideum* genes harboring G4 sequences in their upstream promoter regions can be affected by the addition of a potent G4 ligand, the AuMA porphyrin derivative, indicating that the G4 sequences do play a role in the expression of these genes. We expect that the biological functions of G4 sequences will be more tractable in *Dictyostelium* than in G4-rich organisms such as mammals, allowing to make fundamental discoveries on how G4 formation regulates key biological processes. Our work represents an unprecedented first step to adopt a G4-poor organism as a model for G4 studies.

## Supplementary Material

Supplementary Data
