# Supplementary material for: Mapping and characterization of G-quadruplexes in the genome of the social amoeba Dictyostelium discoideum
Source: Nucleic Acids Res. 2019 Mar 29;47(9):4363–74. doi: 10.1093/nar/gkz196 (PMC6511855; doi:10.1093/nar/gkz196)

Mapping and characterization of G-quadruplexes in the genome of the social amoeba *Dictyostelium discoideum*

Mona Saad^1,2^, Aurore Guédin^1^, Souheila Amor^1^, Amina Bedrat^1^, Nicolas J. Tourasse^1^, Hussein Fayyad-Kazan^2^, Geneviève Pratviel^3^, Laurent Lacroix^4^ & Jean-Louis Mergny^1,5*^

**SUPPLEMENTARY INFORMATION**

**Revised Manuscript – March 8, 2019**

**Supplementary Files**

In addition to this pdf document, additional files are available as supplementary information. They are briefly listed here:

- **dictyG4_script.r**: Describes all the procedure performed in R and the generation of **Figures** **1**, **S1B-C-D**, **S2A-B-C** and **Tables** **1**, **S1A-B**
- **G4HunterFunctionDDAX4.r**: The function used in R for the script "dictyG4_script.r"
- **DDAX4_G4d2019.xlsx**: density and relative density of G4 motifs in *Dictyostelium* with G4Hunter with respect to genomic features compared to the genome average and 1000 reshuffling of the G4 position.
- **Dictyostelium_highly_expressed_UTR5.xls**: Estimated length of 5’ UTR in highly expressed *D. discoideum* AX4 genes. The file gives the location of the transcription start site (TSS) estimated based on RNA-Seq and EST coverage, along with the annotated genomic coordinates of the genes (which actually correspond to the CDS coordinates). The length of the 5’ UTR region is the distance between the TSS and the gene start. For a few genes coverage data revealed that an intron upstream of the annotated gene start has been missed, in which case its length was subtracted to obtain the 5’ UTR length. TSS and UTRs could be estimated for 96 of 162 genes. For the remaining genes, the reason for the lack of estimation is indicated; it is nearly always due to the presence of a fully overlapping antisense RNA (asRNA) gene, which confounds the coverage data.

**Supplementary Methods**

***Estimation of 5’ UTR length***

UTRs are not annotated in the *D. discoideum* genome, which affects the definition of promoter regions. To obtain a rough estimate of the average length of 5’ UTRs in *Dictyostelium* we analyzed the 5’ UTR regions of highly expressed genes for which there is high coverage data. As RNA-Seq coverage data deposited in NCBI turned out to be too sparse, we downloaded raw sequencing data and computed the coverage in-house. We took a set of 52 RNA-Seq datasets from the Shaulsky lab (studies on 24-hour developmental time course; **(1-2)**), mapped the reads onto the genome (using HISAT 2.1.0 **(3)**, run with default parameters except “--min-intronlen 30 --max-intronlen 1000 -k 10 --max-seeds 10”; ~400 million reads mapped), and computed nucleotide coverage (using BEDTOOLS 2.24.0 **(4)**, “genomeCoverageBed” command).

Then, using expression data from these studies available at the dictyExpress website (**(5)**, https://www.dictyexpress.org), we selected the set of 162 most highly expressed protein-coding genes that had an expression value (RPKM) >= 5000 at any time point. Not surprisingly, ~half of them are coding for ribosomal proteins. Finally, we compared the coverage regions with the gene annotations and expressed sequence tags (EST) coverage from dictyBase. We then manually reviewed the data by looking at read/EST mapping and gene structure in the IGV genome browser **(6)**. Manual curation was necessary as for many genes a fully overlapping antisense transcript is predicted (in which case it cannot be determined if the coverage comes from the coding or antisense gene), and for some other genes the annotated start (or EST start) actually corresponds a splice junction, in which case an upstream intron and exon have been missed.

We could estimate the location of the transcription start site (TSS) for 96 genes, based on RNA-Seq and EST coverage (supplementary file “Dictyostelium_highly_expressed_UTR5.xlsx”). The locations obtained from both types of data were usually close (within 50 bp of each other). For RNA-Seq, the coverage decreases gradually towards the 5’ end of the gene and it is a matter of cut-off for deciding where the TSS would be; we have been conservative as to give an upper bound, so the UTR lengths may be slightly overestimated. For these 96 genes, the average 5’ UTR length would be **58 ± 48 bp** (range 2-226) based on EST coverage and **83 ± 49 bp** (range 25-305) based on RNA-Seq coverage. The length is over 100 bp in 25 cases and over 200 bp in only 3 cases.

**Supplementary References**

1. Parikh *et al*. 2010, ***Genome Biology*** 11:R35
2. Rosengarten *et al*. 2015, ***BMC Genomics*** 16:294
3. Kim *et al*. 2015, ***Nature Methods*** 12:357-362
4. Quinlan and Hall 2010, ***Bioinformatics*** 26:841–842
5. Rot *et al*. 2009, ***BMC Bioinformatics*** 10:256
6. Robinson *et al*. 2011*,* ***Nature Biotechnology*** 29:24-26

**Supplementary Tables**

**Table S1a: Very G-rich G4-prones motifs found in Dicty.**

Chromosome Location (nt) Strand ^a^ Sequence ^b^

*chr1 [354933, 354951] - | CCCCCCCCCCCCCCCCCCC*

*chr1 [1692587, 1692614] + | GGGGACAGGGGACAGGGGACAGGGGGGG*

*chr2 [738919, 738940] + | GGGGGGGGGGGGGGGGGGGGGG*

chr2 [2545889, 2545914] - | CCCCTGTACCCCTGTACCCCTCCCCC

*chr2 [4072452, 4072473] - | CCCCCCCCCCCCCCCCCCCCCC*

chr2 [6668513, 6668539] + | GGGGTGGAGGGGTGGGGGTGGGAGGGG

*chr2 [7626286, 7626310] + | GGGGAGGGGAGGGGAGGGGGAGGGG*

chr2 [8324947, 8324971] - | CCCCCACCCCACCCCTCTCGTCCCC

*chr2 [8326099, 8326126] - | CCCCAACCCCAACCCCAACCCCAACCCC*

*chr3 [296857, 296897] - | CCCCCTTCCCCCTTCCCCCTTCCCCCTTCCCCCTTCCCCCC*

*chr3 [1611836, 1611863] + | GGGGTTGGGGTTGGGGTTGGGGTTGGGG*

chr3 [1907066, 1907088] + | GGGGGTGGGGGTGGGGTAAGGGG

chr3 [5185454, 5185478] + | GGGGTTGGGGTAATTGGGGTTGGGG

*chr3 [5394992, 5395022] + | GGGGTTGTTGGGGTTGTTGGGGTTGTTGGGG*

chr4 [3759614, 3759635] + | GGGGAGGGGTAGGGGGAGGGGG

chr4 [4399659, 4399677] + | GGGGTGGGGTGGGGAGGGG

chr5 [1814739, 1814760] - | CCCCCCCCCCCCCCCCTCCCCC

*chr5 [2677402, 2677423] - | CCCCAACCCCAACCCCAACCCC*

*chr5 [4800459, 4800477] - | CCCCCCCCCCCCCCCCCCC*

chr6 [458691, 458710] + | GGGGGGGGGGGGGGAAGGGG

chr6 [1152079, 1152105] + | GGGGGGGGCAAAGGGGGGGGGGGGGGG

*chr6 [1290182, 1290209] - | CCCCAACCCCAACCCCAACCCCAACCCC*

chr6 [2790736, 2790775] + | GGGGGGGAGGTTGGGGGGGTTTTGGGGGCCCAAAAGGGGG

^a^ “+“ strand means the sequence corresponds to the G-rich motif; “-“ means the complementary sequence is prone to G4 formation.

^b^ Sequences in italic correspond to repeats of G (or C)-rich motifs.

**Table S1b: Longest G4 motifs found in Dicty**

chr1 [1040148, 1040211]

GTTGTTGTTGGGGTTGGGGTTGGGGTTGTTGTTGTTGTTGGGGCTGGGGTTGGGGTTGAAAGTG

chr3 [5394989, 5395064]

GTTGGGGTTGTTGGGGTTGTTGGGGTTGTTGGGGTTGTTGTTGGGGTTGTTGGGGTTGTTGTTGGGGTTGTTGGGG

chr5 [2677390, 2677441]

CACCCAATCCAACCCCAACCCCAACCCCAACCCCAACTCCAACCCCAACCCC

chr6 [3364846, 3364948]

GTTGGTAGGGGGGGTGGTGGGGGTAGTGAGAGGGAGGAGGTGGGGAGAGTGTGGGGGTGTGGGGTGTATTTTGAGGAGGGGAGGGGAGGTGGGAAAGGTGAGG

chr6 [3365020, 3365085]

GTAAGGGGGGATGGAAGGGGTGGGGGGGTTTGGTGGAAGAAGGGGGGGGGAGAGGAATTTTTGGGG

**Supplementary Figures**

**Figure S1.** *(Next pages)*

**(A)** Global G4FS density obtained with G4Hunter at different thresholds for the human (hg19, blue circle) and *D. discoideum* (Dd *low* (threshold ≤1.4) = diamonds and Dd *high* (threshold >1.4) = purple crosses) genomes. Lines correspond to exponential fit of the data.

**(B)** G4FS density for each chromosome (1 to 6) of the *D. discoideum* genome at different thresholds (1.2, 1.5, 1.75, 2). The p-values for chromosome 6 were calculated using the mean and standard deviation of the density for the six chromosomes for each threshold. The dotted lines represent the global G4FS density for the whole genome at the given threshold.

**(C)** Density and coverage for the transcripts on the different chromosomes.

**(D)** *(next page)* GC content in promoters (500bp) according to the presence or the absence of a G4FS at threshold 1.5, 1.75 and 2.


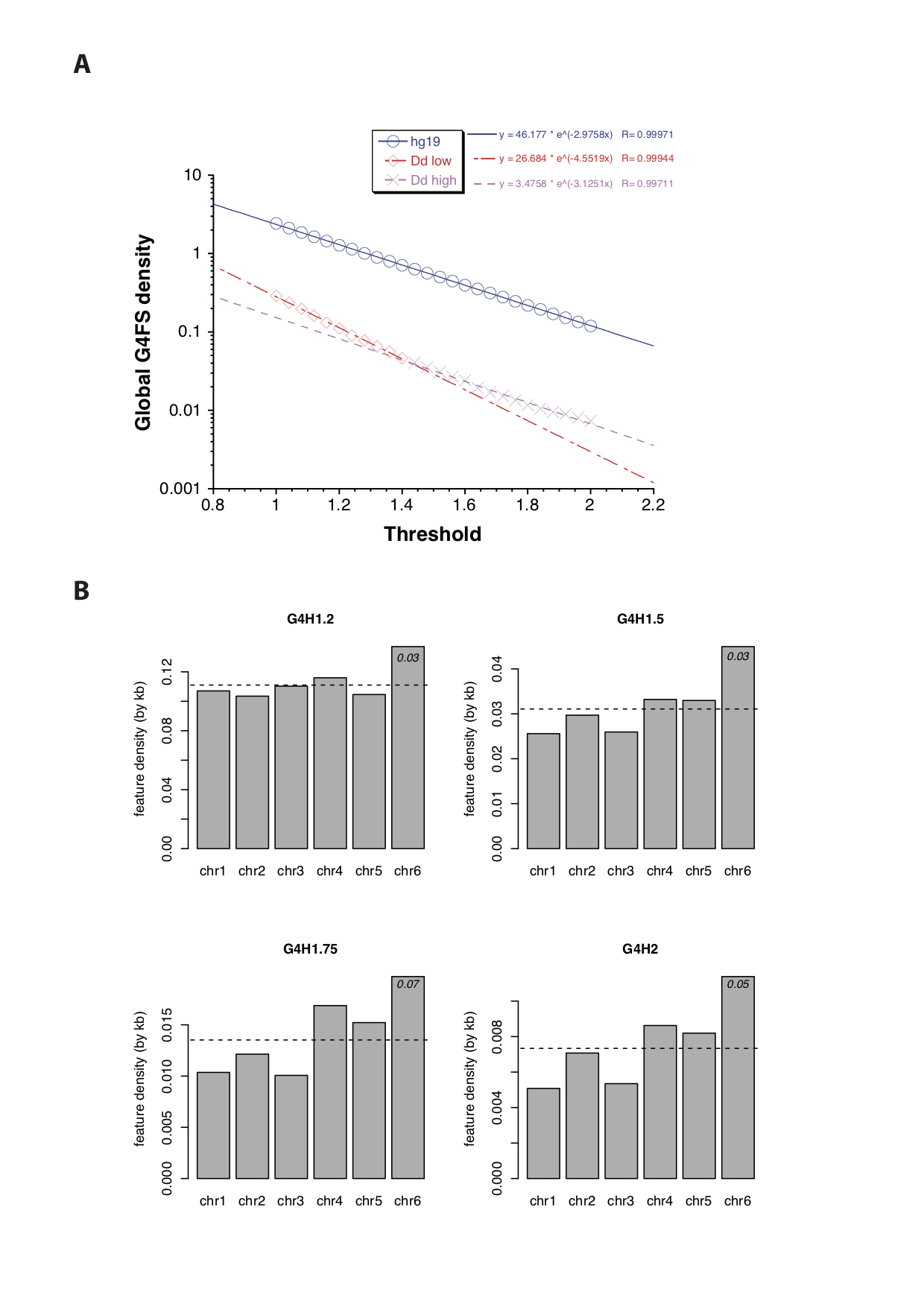


**
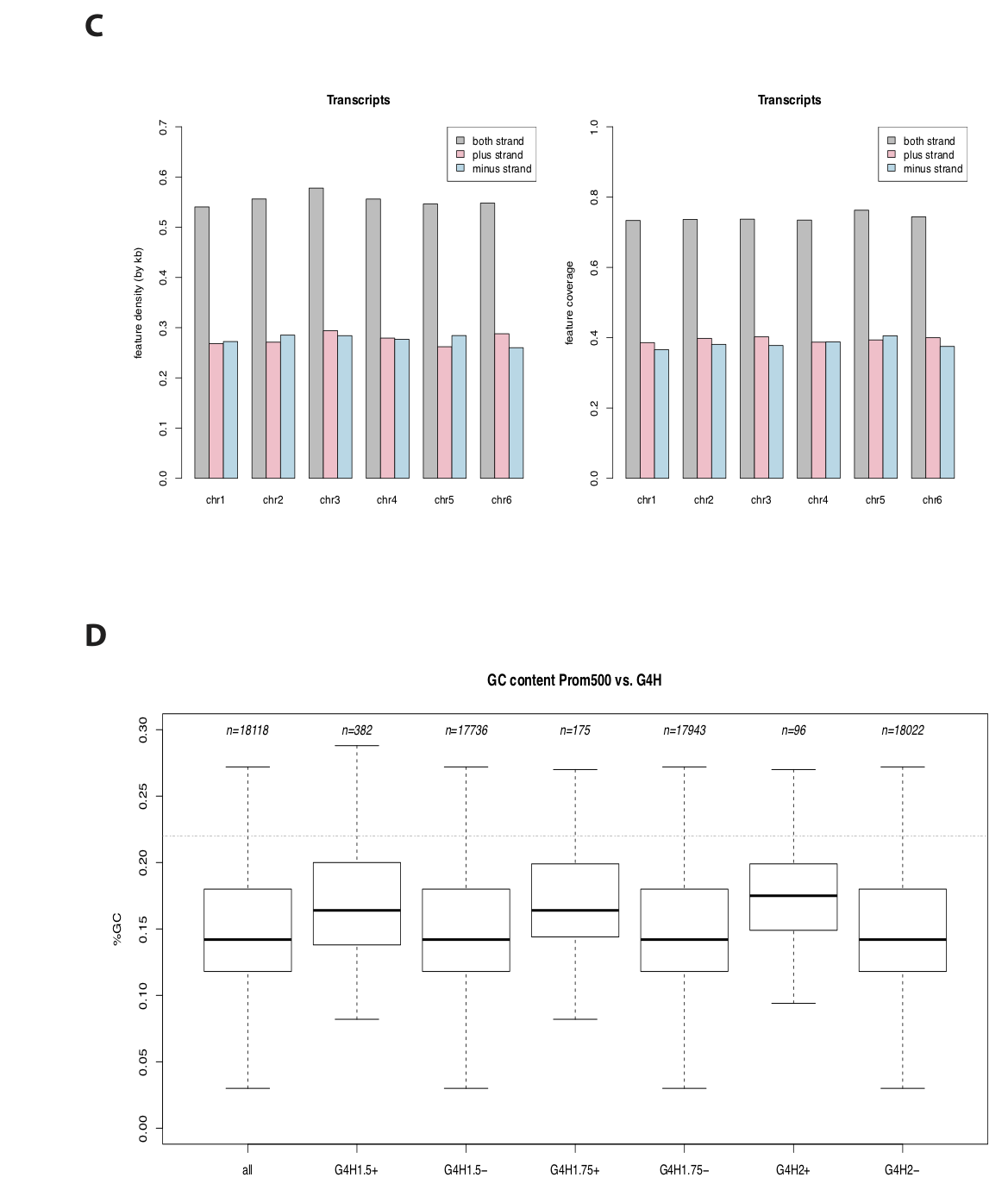
**

**Figure S2.** *(see next page)* **(A)** G4FS density as in Figure 1A but after taking into account the strand information of the features. For promoters, the strand of the corresponding transcript was used. Dark color= coding strand, light color=non-coding strand. The dotted line indicates the average G4FS density for the two strands of the genome. **(B)** Profile of G4FS around exon/intron junctions for a threshold of 1.5. Red = coding strand; blue = non-coding strand. Shaded areas around the curves represent the 95% confidence interval (CI95) based on the distribution of the fraction of G4FS at this position for all the junctions. **(C)** Distribution of G content on both strands for the different genomic features represented as boxplot. For promoter, the strand of the corresponding transcript was used. Dark color = coding strand, light color = non-coding strand. The dotted line indicates the average G content of the genome.


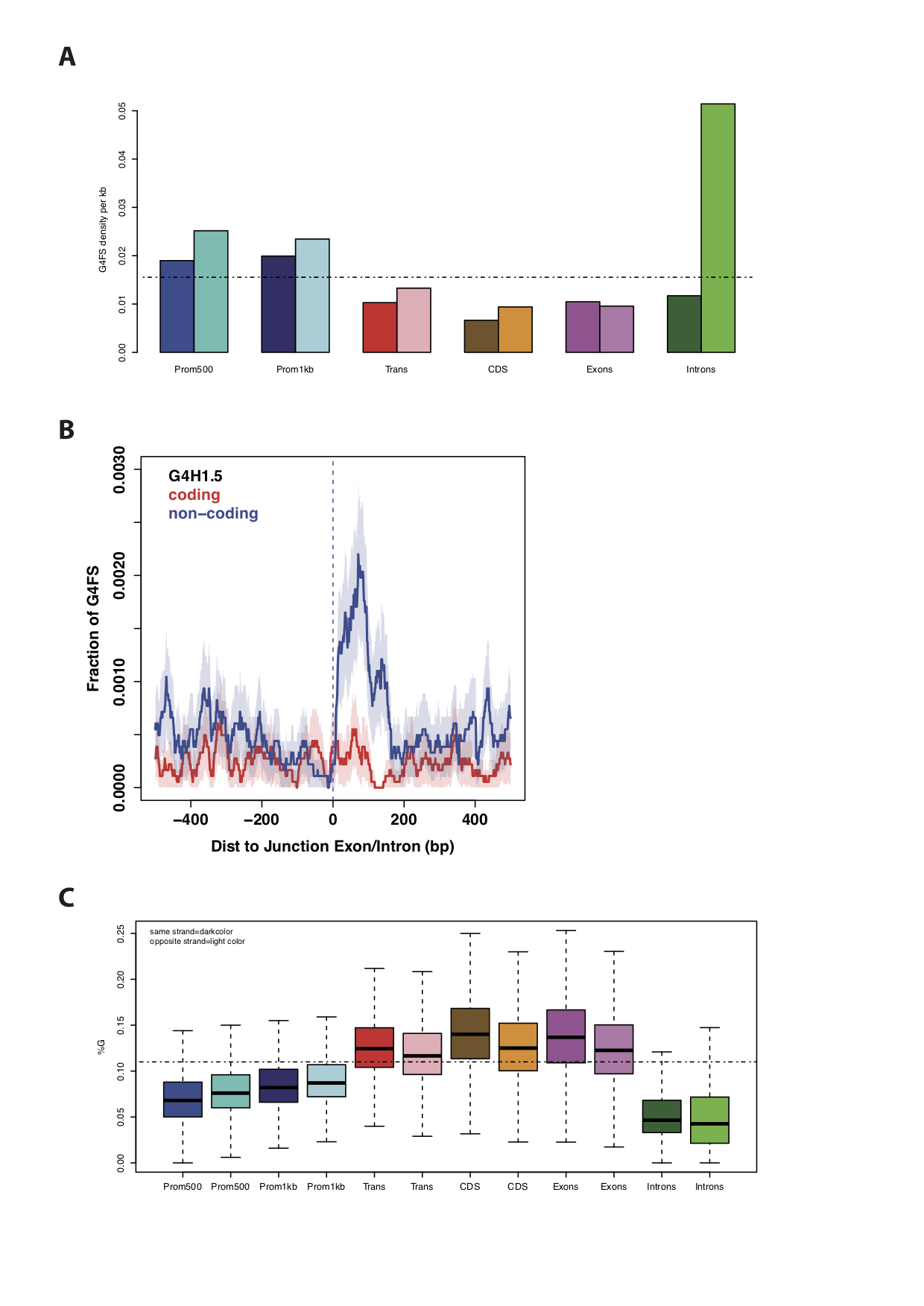


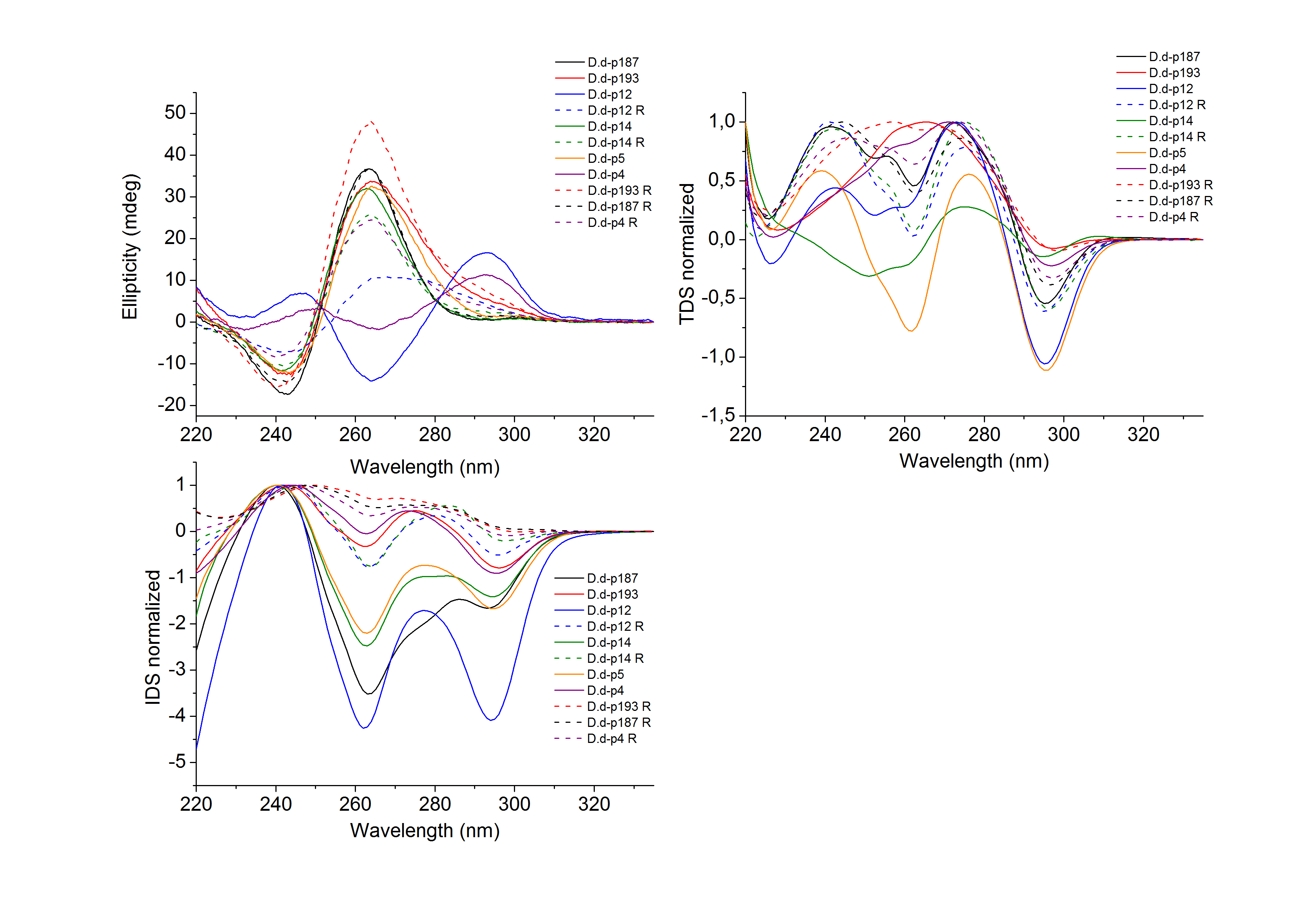
**Figure S3. (A)** CD spectra recorded at 25°C. **(B)** Thermal difference spectra (TDS). **(C)** Isothermal difference spectra (IDS) at 25°C. Except for **p172**, which was folded in 10 mM KCl, all DNA samples were folded in 100 mM KCl at 4 µM. RNA samples were folded in 50 mM KCl. TDS and IDS spectra were normalized to [0;1].

**Figure S4.** UV melting and annealing traces recorded at 295 nm after baseline subtraction at 335 nm. The DNA samples were folded in 100 mM KCl at 4 µM and the RNA samples were folded at 50 mM KCl.


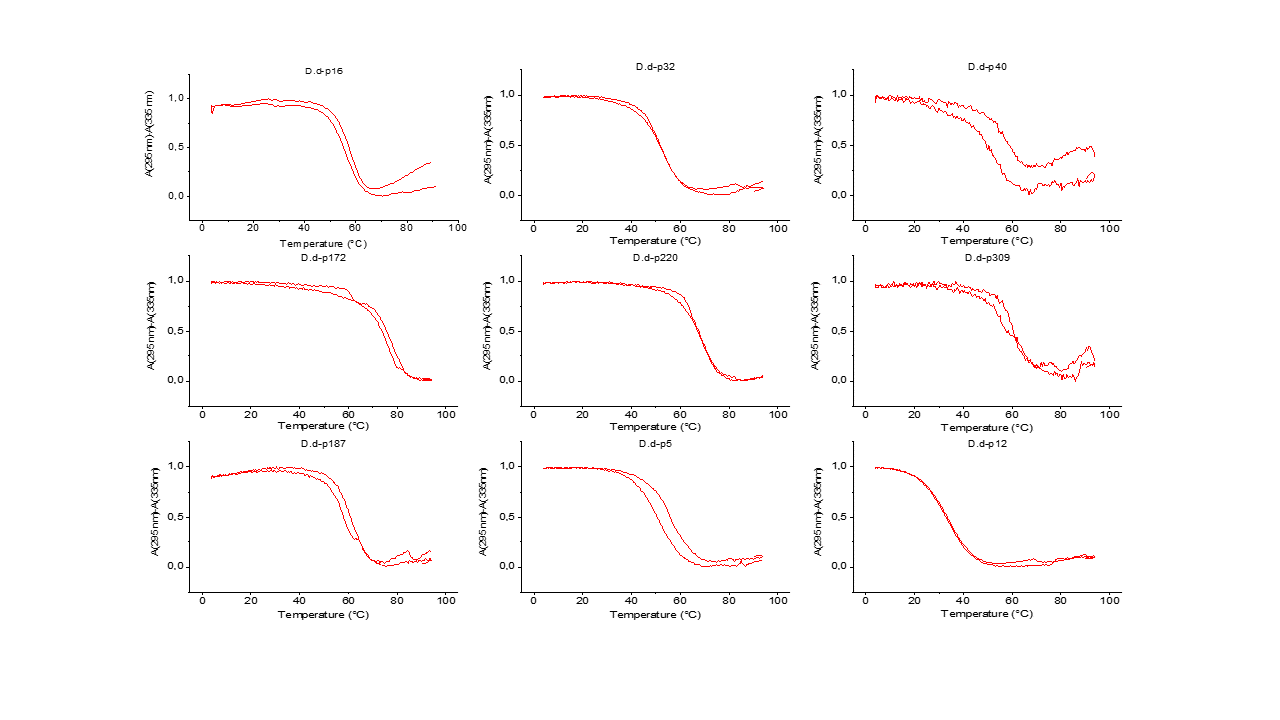


**Figure S4.** *continued*


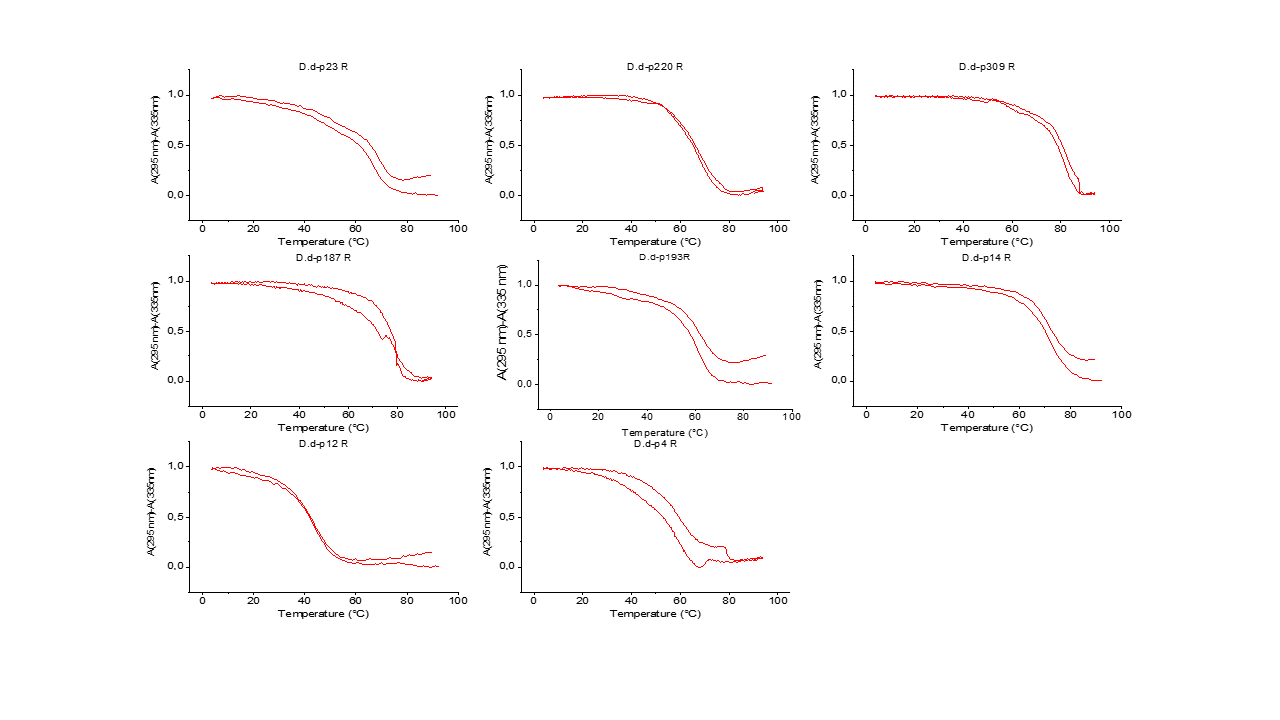


**Figure S4.** *continued*


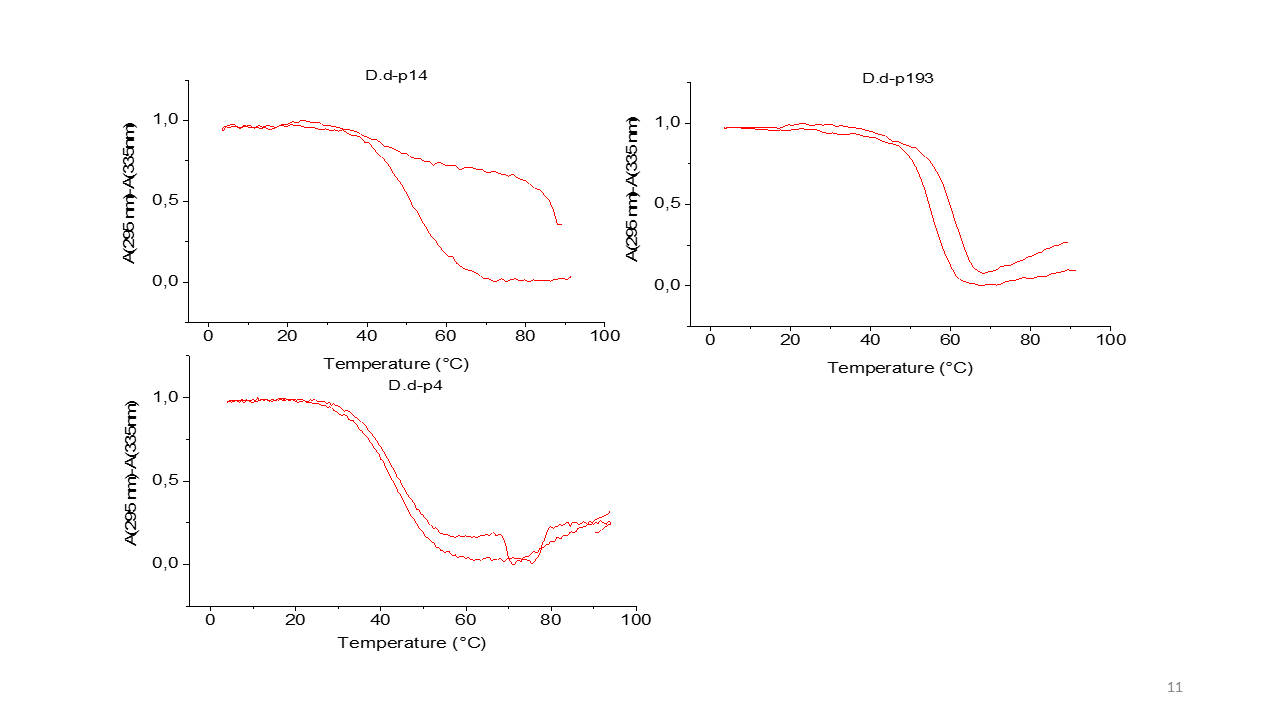


**Figure S5.** Imino proton spectra in 20 mM potassium phosphate buffer at pH 6.9 with 70 mM KCl at 25°C.


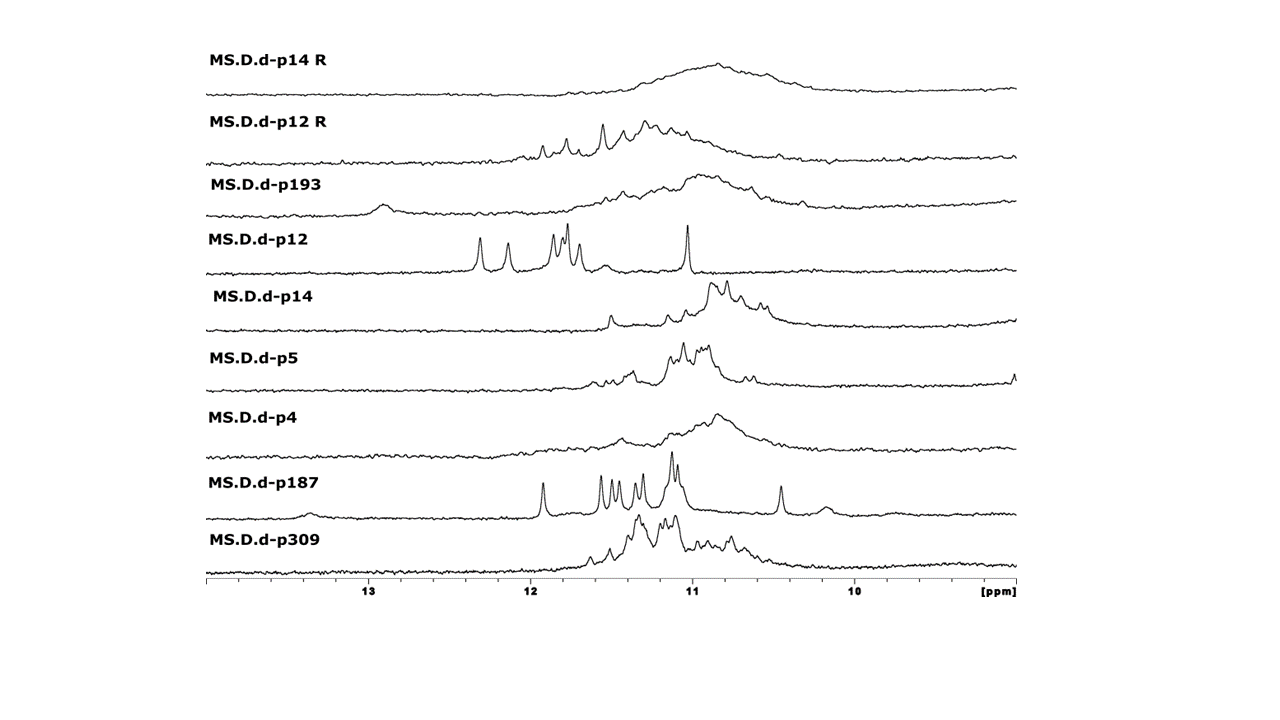


**Figure S6.** G4 structures detected on nondenaturing polyacrylamide gel electrophoresis by “Stains-All” staining. Twenty-one different oligonucleotides were loaded at 100 and 30 µM concentrations on a nondenaturing 15% acrylamide gel supplemented with 10 mM KCl. Migration was performed at 20°C. Migration markers were single-stranded dTn (n= 9, 15 or 21; note that the diffuse bands below correspond to the dyes Bromophenol blue and Xylene cyanol used to follow migration). After electrophoresis, the gels were visualized by MF-ChemiBIS 3.2 bioimaging system.


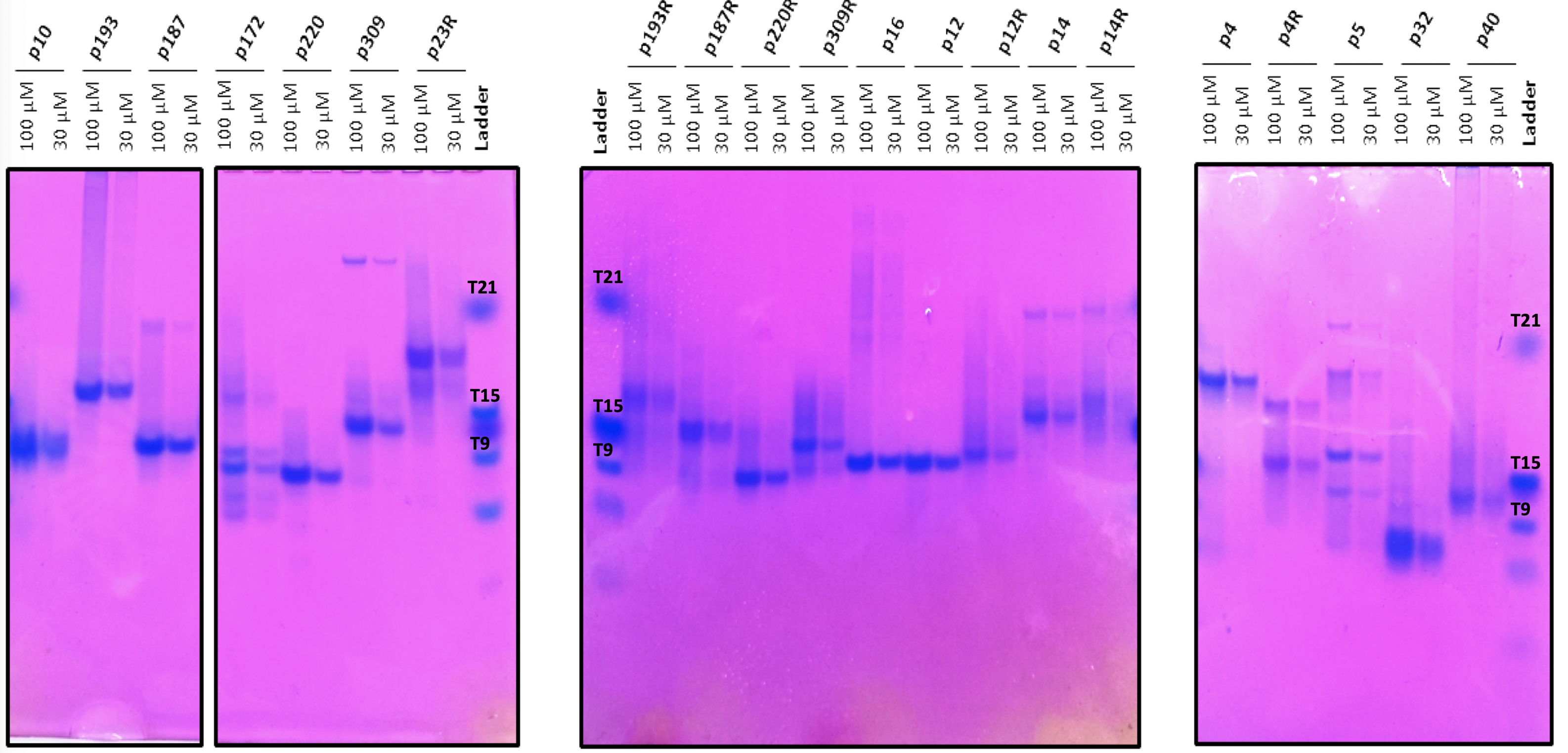


**Figure S7.** FRET melting demonstrates the stabilizing effect of AuMA for F21T in presence of large excess of *Dictyostelium* sequences **p10**, **p187**, **p193**, **p32** and **p40**. AuMA was used at 0.5 µM. F21T and Dicty sequences were folded at 0.2 µM in 10 mM KCl combined with 90 mM KCl in the presence of 10 mM LiCaco buffer. The competitors were used at two different strand concentrations (3 and 10 µM).


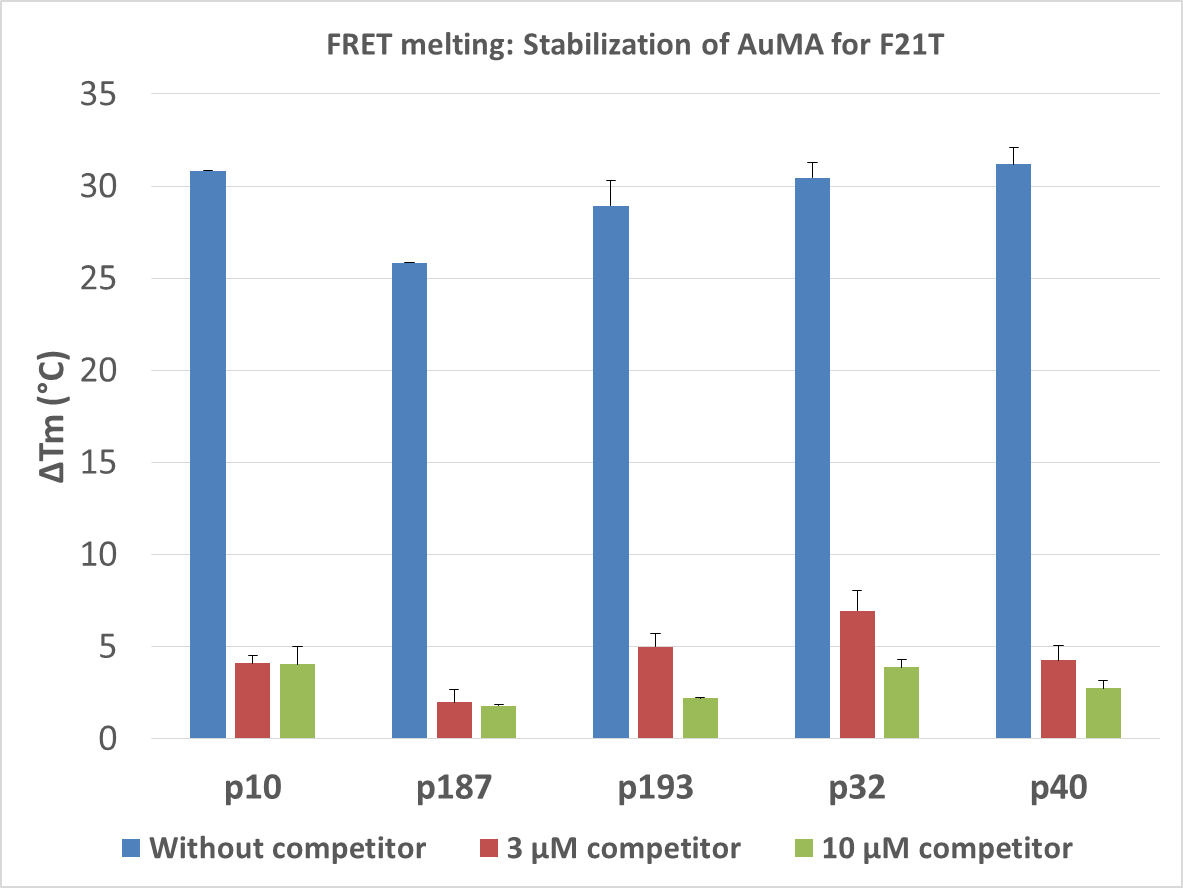


**Figure S8.** Distribution of 5’ UTR length for 96 highly expressed *D. discoideum* genes (gene list provided in Supplementary file “Dictyostelium_highly_expressed_UTR5.xlsx”). UTR length was estimated based on either RNA-Seq (top) or EST (bottom) coverage.


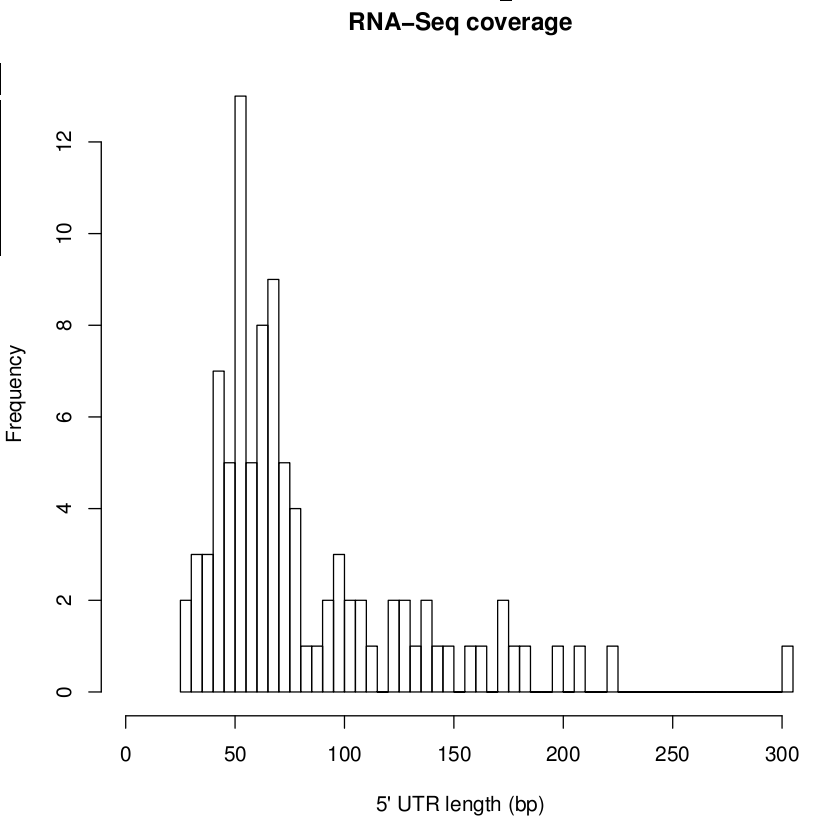


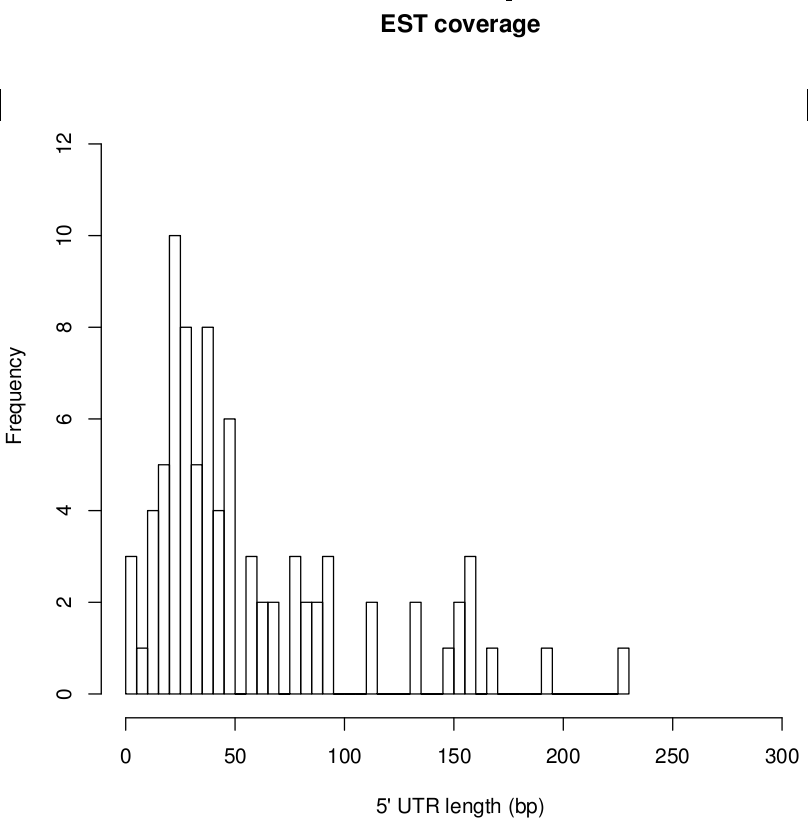

Supplement: Supplementary Data [file gkz196_supplemental_files.zip › 2019-03-08_Saad_SIv2.docx]
